# Metabotyping of 30 maize hybrids under early-sowing conditions reveals potential marker-metabolites for breeding

**DOI:** 10.1007/s11306-018-1427-8

**Published:** 2018-09-26

**Authors:** Nadia Lamari, Vanessa Zhendre, Maria Urrutia, Stéphane Bernillon, Mickaël Maucourt, Catherine Deborde, Duyen Prodhomme, Daniel Jacob, Patricia Ballias, Dominique Rolin, Hélène Sellier, Dominique Rabier, Yves Gibon, Catherine Giauffret, Annick Moing

**Affiliations:** 1 UMR1332 Biologie du Fruit et Pathologie, INRA, Univ. Bordeaux, Centre INRA de Nouvelle Aquitaine - Bordeaux, 71 av Edouard Bourlaux, 33140 Villenave d’Ornon, France; 2 Plateforme Métabolome du Centre de Génomique Fonctionnelle Bordeaux, MetaboHUB, IBVM, Centre INRA de Nouvelle Aquitaine - Bordeaux, 71 av Edouard Bourlaux, 33140 Villenave d’Ornon, France; 3 INRA, UR AgroImpact, Estrées-Mons, 80203 Péronne, France; 4 INRA UE GCIE, Estrées-Mons, France; 5 Present Address: Earth Institute, O’Brien Centre for Science, School of Biology and Environmental Science, University College Dublin, Belfield, Dublin, Ireland; 6 Present Address: Enza Zaden Centro de Investigacion S.L., 04710 Santa Maria del Aguila, Almería, Spain

**Keywords:** Chilling tolerance, Environmental changes, Maize, Marker metabolites, Metabolic distance, Metabolomics

## Abstract

**Introduction:**

In Northern Europe, maize early-sowing used to maximize yield may lead to moderate damages of seedlings due to chilling without visual phenotypes. Genetic studies and breeding for chilling tolerance remain necessary, and metabolic markers would be particularly useful in this context.

**Objectives:**

Using an untargeted metabolomic approach on a collection of maize hybrids, our aim was to identify metabolite signatures and/or metabolites associated with chilling responses at the vegetative stage, to search for metabolites differentiating groups of hybrids based on silage-earliness, and to search for marker-metabolites correlated with aerial biomass.

**Methods:**

Thirty genetically-diverse maize dent inbred-lines (*Zea mays*) crossed to a flint inbred-line were sown in a field to assess metabolite profiles upon cold treatment induced by a modification of sowing date, and characterized with climatic measurements and phenotyping.

**Results:**

NMR- and LC-MS-based metabolomic profiling revealed the biological variation of primary and specialized metabolites in young leaves of plants before flowering-stage. The effect of early-sowing on leaf composition was larger than that of genotype, and several metabolites were associated to sowing response. The metabolic distances between genotypes based on leaf compositional data were not related to the genotype admixture groups, and their variability was lower under early-sowing than normal-sowing. Several metabolites or metabolite-features were related to silage-earliness groups in the normal-sowing condition, some of which were confirmed the following year. Correlation networks involving metabolites and aerial biomass suggested marker-metabolites for breeding for chilling tolerance.

**Conclusion:**

After validation in other experiments and larger genotype panels, these marker-metabolites can contribute to breeding.

**Electronic supplementary material:**

The online version of this article (10.1007/s11306-018-1427-8) contains supplementary material, which is available to authorized users.

## Introduction

Temperature is a key factor for plant development and productivity, especially for plant crops including cereals, which are important sources of food and animal feed. Maize is a cereal of great economic importance, with a grain world production of about 1022 million tons in 2014 (faostat3.fao.org, FAO food and agriculture data). In Northern Europe, maize early-maturing hybrids are usually sown from mid-April to early-May and are harvested in mid-September for silage or mid-October for grain. Early sowing is considered to be a way of maximizing yield of these early maturing hybrids. Breeding schemes of maize early hybrids in Europe has long been relying on the flint Lacaune genetic pool and a complementary dent pool showing a high combining ability with this flint group. Most often, early sowing leads to moderate damage of seedlings due to chilling non-freezing temperatures, without clear visible phenotype in the field because of the cold tolerance of the flint parental line. But recently, medium late or late germplasm (such as Iodent, BSS and Lancaster) has been introduced into the flint pool (Barrière et al. 2006). This late material, bred in warmer areas, has not been selected for chilling tolerance and is likely to bring a higher susceptibility to low temperatures.

With less chilling-tolerant hybrids in such extreme environments, chilling stress may induce symptoms at the plant level such as leaf chlorosis, development and growth retardation, but also shoot or root tissue injury with necrosis or even death (Greaves 1996). Part of these symptoms may be related with reduced photosynthesis and photoinhibition occurrence. Chilling may also induce modifications at the cellular level such as osmotic stress, membrane disorganization, low protein activity and possibly an increase in reactive oxygen species (ROS), with corresponding metabolic changes in relation with the general reduction of enzyme activities and reconfiguration of the metabolic network (Krasensky and Jonak 2012). Thus, there is a renewed interest for cold tolerance in breeding schemes. Among tools that have to be developed to help genetic studies and further breeding for early-sowing tolerance, including chilling tolerance, metabolic markers representative of cold resistance would be particularly useful.

During chilling acclimation, the complex reprogramming of gene expression leads to the accumulation of protective proteins and also protective metabolites such as compatible solutes (including soluble sugars, sugar alcohols, proline and betaine) or ROS protectors, and also signalling metabolites (Zhu et al. 2007). Therefore, metabolomics appears as a method of choice for precise phenotyping. Plant metabolomics studies often combine several analytical strategies (Hall 2011). Gas chromatography coupled with mass spectrometry and proton NMR spectroscopy (^1^H-NMR) of polar extracts give access to primary metabolites. Liquid chromatography coupled with mass spectrometry (LC-MS) of semi-polar extracts provides relative quantification of secondary metabolites belonging to diverse families of compounds including flavonoids, hydroxycinnamates and benzoxazinoids. Such analytical approaches have been largely used recently for cereals (Balmer et al. 2013; Khakimov et al. 2014), including maize kernels, leaves or other organs for genomics or studies of environmental effects (e.g. Amiour et al. 2012; Baniasadi et al. 2014).

Besides primary metabolites (Cañas et al. 2017; Sun et al. 2016b), the maize leaf contains several families of specialized metabolites, several of which have been shown to be implicated in biotic stresses such as the quinic acid derivative chlorogenic acid (Cortés-Cruz et al. 2003), the flavone glycoside maysin (Rector et al. 2003), and benzoxazinoids (Frey et al. 1997; Meihls et al. 2013). The use of metabolomics to understand the response of plants including crops to particular environments has largely been used (Obata and Fernie 2012; Arbona et al. 2013), as for instance recently in potato facing drought (Sprenger et al. 2016) or maize subjected to variations in temperature (Sun et al. 2016a). It has also been used to predict agronomic important phenotypes of plants for potato grown in different environments (Steinfath et al. 2010) and for maize hybrids (Riedelsheimer et al. 2012a, b).

Compositional data from metabolomics approaches combined with chemometrics have also been largely used for distinguishing the geographical and variety origin of several foods (Cubero-Leon et al. 2014), including those from plants. Metabolomics data can be used for establishing a measure of the metabolic distance between accessions in a given environment, and across environments. In a study involving nine accessions of Arabidopsis, a minor correlation was shown between genetic and metabolic diversity (Houshyani et al. 2012) for this model species. A study of three rice cultivars showed that metabolomic diversity of grain was highly associated with the genetic distance between these varieties (Calingacion et al. 2012). Similarly, a study of six soybean varieties demonstrated that metabolomic data on seed could be correlated with genotypic data (Kusano et al. 2015). In maize, a study of 19 lines showed that metabolite accumulation in leaves mostly depended on the genetic background (Cañas et al. 2017). A metabolomic study of grain involving larger sets of maize genotypes showed that subpopulations could be differentiated in a way consistent with the genetic variation of these lines (Venkatesh et al. 2016).

Using an untargeted metabolomic approach on a collection of 30 genetically-diverse maize hybrids cultivated in the field, the aim of the present study was to identify metabolite signatures and/or metabolites associated with severe chilling vs mild chilling responses of maize plants at the vegetative stage (8-visible-leaf stage). The compositional data were also used to measure phenotypic distances between genotypes, and search for metabolites differentiating groups of hybrids based on silage-earliness. Finally, these compositional data were combined with aerial biomass data to search for candidate marker-metabolites correlated with this agronomical trait. When such associations are confirmed with another experiment, they can be considered as marker metabolites that could be measured in large panels for selection purposes.

## Materials and methods

### Plant material, growth conditions and sampling

Thirty genetically diverse dent maize inbred-lines (*Zea mays* ssp. *mays*, Table 1) structured into four admixture groups (European, Iodent, Lancaster, Stiff Stalk (Ganal et al. 2011; Rincent et al. 2014)) were selected according to their diversity based on pedigree, genotyping and flowering dates. They were crossed to the flint inbred-line UH007 (Univ. Hohenheim, Germany) developed to improve combining ability with Iodent and Stiff-Stalk lines for earliness, yield of grain and stover, and sown as hybrids in a field located at Estrées-Mons (Northern France, 49°52′44″N, 3°0′27″E). The hybrids were classified into five earliness groups according to their silage earliness evaluated through the mean dry matter content at harvest in a European field experimental network (Rincent et al. 2014). The groups were defined as in the French variety registration protocol, with a difference of 3% in dry matter content between hybrids from two successive groups. Konfians (KWS, Champhol, France), a commercial hybrid corresponding to the mean silage-earliness of the 30 hybrids, was also grown in the field for the preparation of samples dedicated to spectra annotation. The soil was a deep loam. N:P:K fertilizer and irrigation were applied. Two sowing dates corresponding to two “conditions” were used: early-sowing (ES) on 15 April 2013 and normal-sowing (NS) on 13 May 2013. The air temperature, rainfall, air vapour pressure deficit, and global radiation were recorded by a meteorological station located at approximately 600 m from the field (Online Resource 1). Thermal times starting from emergence (equivalent to the number of equivalent days at 20 °C) were calculated from air temperature (Parent and Tardieu 2012) for each condition. The experimental design for each condition consisted in four individual rows of 40 plants per genotype, planted in three randomized blocks. For each hybrid and block, the youngest ligulated leaf (usually from the third to the fifth leaf depending on the genotype) at the 8-visible-leaf plant stage was harvested between the times of 10:00 and 13:00 from 10 plants per block. A single central 5-cm section of each leaf without the main vein was selected, pooled and immediately frozen in liquid nitrogen. Each biological replicate corresponded to a block. ES samples were harvested on 14 June 2013 (18.4 equivalent days at 20 °C after emergence, d_20 °C_) and NS samples on 5 July 2013 (20.1 d_20 °C_).

**Table 1:** List of the 30 maize hybrids of the dent panel selected based upon their diversity

Accession	Origin	Admixture group	Silage-earliness group
A374_inra	USDA	European	Mid-early
B89_inra	USDA	European	Mid-early
EZ11A_csic	CSIC	European	Very late
F7028_inra	INRA	European	Very-early
FV252_inra	INRA	European	Very-early
MS153_inra	USDA	European	Late
Oh02_inra	USDA	European	Mid-early
Oh33_inra	USDA	European	Early
W117_inra	USDA	European	Early
D06_uh	UH	Iodent	Very-early
D09_uh	UH	Iodent	Very-early
F912_inra	INRA	Iodent	Early
FC1890_inra	INRA	Iodent	Early
FV353_inra	INRA	Iodent	Early
PH207_usda	USDA	Iodent	Mid-early
UH304_uh	UHOH	Iodent	Early
B100_uh	USDA	Lancaster	Late
B97_inra	USDA	Lancaster	Late
LH38_usda	USDA	Lancaster	Mid-early
Mo17_inra	USDA	Lancaster	Late
W64A_inra	USDA	Lancaster	Mid-early
B104_inra	USDA	Stiff stalk	Very-late
B105_inra	USDA	Stiff stalk	Late
B73_inra	USDA	Stiff stalk	Late
B84_inra	USDA	Stiff stalk	Late
EC169_ciam	CIAM	Stiff stalk	Early
EZ37_csic	CSIC	Stiff stalk	Mid-early
F1808_inra	INRA	Stiff stalk	Mid-early
F618_inra	INRA	Stiff stalk	Mid-early
FR19_usda	USDA	Stiff stalk	Mid-early

Accession indicates the female inbred common name followed by the origin of the seed lot used for the project. The male tester line was UH007. Admixture groups are based on Panzea SNPs from Illumina MaizeSNP50 BeadChip (Ganal et al. 2011)

Hybrids were classified into five earliness groups based on the dry matter content of aerial biomass at silage harvest in a European network

*USDA* United States Department of Agriculture, USA, *CSIC* Consejo Superior de Investigaciones Científicas, Spain, *UH* Universität Hohenheim, Germany, *CIAM* Centro Investigacións Agrarias de Mabegondo, Spain

Snap-frozen leaf samples (about 2 g plunged into liquid nitrogen within seconds after sampling) were stored at − 80 °C until grinding in liquid nitrogen (2010 Geno/Grinder, Spex, Stanmore, UK). Ground samples were stored at − 80 °C before freeze-drying that allowed measurement of the dry weight over fresh weight ratio. Lyophilized samples were kept at − 20 °C in dry atmosphere before metabolomic analyses.

### Plant phenotyping

The leaf number was recorded on eight plants per genotype and block on the day before, or the day of, leaf sample harvest for metabolomic analyses. Chlorophyll fluorescence measurements (variable-fluorescence over maximal-fluorescence ratio, Fv/Fm) were performed on the youngest ligulated leaf of four plants per block using a rapid screening chlorophyll fluorimeter (Pocket PEA, Hansatech, Norfolk, U.K.) to estimate the maximum quantum efficiency of photosystem II (after a minimum 20 min of dark adaptation) and check for the occurrence of photoinhibition (Maxwell and Johnson 2000). The days of fluorescence measurements were chosen according to field weather data (low temperature and high light) on 4 June 2013 for ES and 26 June 2013 for NS. For biomass determination, 24 plants per genotype (eight plants per block, different from the 10 plants used to collect leaf samples) were harvested 4–5 days after the harvest of leaf samples for metabolomic analyses, on 19 June 2013 and 9 July 2013 for the ES and NS conditions, respectively. The dry masses of the aerial part were measured after drying at 80 °C in an oven until constant weight. Because of the cool and wet season, it was impossible to reach physiological maturity (around 32% grain moisture content) by the end of October neither for NS nor ES. Therefore, 18 other plants per genotype were harvested around 40% grain moisture content (half milk line stage) in early November (4–5 November for ES, 7–8 November for NS) for final biomass and grain yield estimation. For all traits, least square means (ls-means) per genotype were calculated using ASReml-R v3.0 software (Gilmour et al. 2009) with genotype and block as fixed effects.

### ^1^H-NMR analysis of polar metabolites

For NMR analysis of leaf samples, polar metabolites were extracted from 20 mg DW using a hot ethanol/water series and quantified by ^1^H-NMR as previously described (Biais et al. 2009) with special care to allow absolute quantification of individual metabolites as detailed in Online Resource 2. A 90° pulse angle and an electronic reference with calibration curves for quantification were used. ^1^H-NMR spectra of Konfians commercial hybrid cultivated in NS and ES conditions were converted into JCAMP-DX format and have been deposited, with associated metadata, into the Metabolomics Repository of Bordeaux MeRy-B (http://services.cbib.u-bordeaux.fr/MERYB/res/project/M13001). The assignments of metabolites in the NMR spectra (Online Resource 3) were made by comparing the proton chemical shifts with literature (Fan 1996; Mounet et al. 2007) or database values (MeRy-B 2011, http://bit.ly/meryb; HMDB, http://www.hmdb.ca/), by comparison with 1D and 2D spectra of authentic compounds recorded in the same solvent conditions (in-house library) and by spiking the samples. ^1^H-^1^H COSY and ^1^H-^13^C HSQC 2D NMR experiments were acquired for selected samples for assignment verification.

### Starch measurement

Starch contents of leaf samples were determined in the previously obtained pellets (see above and Online Resource 2) after polar compound extraction (Hendriks et al. 2003), using 96-well polystyrene microplates (Sarstedt, Marnay, France) and expressed in glucose equivalents. Extractions and assays were performed using a robotised Starlet platform (Hamilton, Villebon sur Yvette, France) and absorbencies were read at 340 nm using MP96 readers (SAFAS, Monaco).

### LC-ESI-QTOF-MS analysis of semi-polar metabolites

Lyophilized maize samples (20 ± 0.5 mg DW) from the field experiment were extracted with 1 mL of methanol/water (70/30, v/v) with 0.1% formic acid and methyl vanillate as internal standard to check if injection was performed correctly by the autosampler. The methanol/water with 0.1% formic acid extracts were injected without any other preparation step and analysed by LC-QTOF-MS as detailed in Online Resource 2. Chromatographic runs were conducted using a reverse phase column. Mass spectral analyses were performed with a hybrid quadrupole/time-of-flight mass spectrometer (micrOTOF-*Q*, Bruker Daltonics, Bremen, Germany) equipped with an electrospray ionization (ESI) source. The data was processed using Workflow4Metabolomics in the Galaxy environment (Giacomoni et al. 2015) as detailed in Online Resource 2. This resulted in a high quality dataset retaining 2839 ions over the 7307 initial ones and used for further statistical analyses. Since maysin peak was saturated, it did not pass the ANOVA filtration step. Molecular formulae were generated using SmartFormula software (Bruker, Bremen, Germany). The final annotation and putative name assignments (Online Resource 4) were also achieved by comparing with MS-related information in the literature (Fridén and Sjöberg 2014; Gomez-Roldan et al. 2014; Gómez-Romero et al. 2010; Marti et al. 2013; Walker et al. 2011) and databases (MassBank, MoNA, mzCloud).

### Data analyses

Absolute or relative metabolite contents expressed on a DW basis were used for statistical analyses. Principal component analysis (PCA, correlation matrix) and Volcano plot analysis were performed using R scripts in BioStatFlow web application (biostatflow.org, v2.7.7) to visualize the global effect of sowing condition, or look for metabolites or metabolite features affected by the sowing condition and considered as response-markers of the sowing condition. For the latter metabolites or features, ANOVAs for genotype effect were performed on log2 transformed data (*P* < 0.05) for each culture condition separately. Euclidian distance matrices of compositional data (ls-means of biological replicates per genotype) mean-centred and scaled to unit variance were constructed using Multi Experiment Viewer (Saeed et al. 2003). Orthogonal signal correction partial least-square discriminant analyses (OSC-PLS-DA) were performed with BioStatFlow on genotype ls-means to search for metabolites or metabolite signatures linked with silage-earliness groups. After unit-variance scaling, a model with two components was used with 500 permutations for cross validation. The metabolites or metabolite signatures highlighted with OSC-PLS-DA were checked using variance analysis on log10-transformed data followed with Tukey’s studentized test performed with BioStatFlow. To visualize metabolite or metabolite-biomass coregulations, correlation networks based on Pearson correlations after log2 transformation of all data and with a *q* < 0.02 threshold after FDR correction were calculated and visualized using Fruchterman layout in BioStatFlow and Cytoscape software version 3.5 (Shannon et al. 2002; http://www.cytoscape.org/).

## Results

### Early sowing affects plant early growth but not final aerial biomass

The environmental variables (Online Resource 5) showed that the mean air temperature was similar for the two sowing conditions from sowing to emergence (10.8 and 10.4 °C for ES and NS, respectively), and lower by 3.8 °C for the ES condition for the period ranging from emergence to the 8-leaf stage that also lasted longer for this condition. The mean global radiation was similar for the two conditions from emergence to 8-leaf stage. For the ES condition, 83% of days between emergence and sample harvest had a temperature below 15 °C, and this percentage dropped to 36% for the NS condition.

Leaf fluorescence measurements allowed verifying for the amount of photoinhibition of plants after the strongest photoinhibitory period recorded between emergence and sampling for each sowing condition. This period occurred after 4 days at 11.9 °C with a mean daily global radiation of 2329 J/cm^2^ for ES, and after 2 days at 13.8 °C with a mean daily global radiation of 2329 J/cm^2^ for NS. The mean ± SD of fv/fm values were 0.57 ± 0.03 and 0.63 ± 0.04 for ES and NS respectively (n = 30 genotypes), with no significant differences (*P* > 0.05) between genotypes.

Developmental stages at leaf and plant harvests for biochemical and biomass analyses, respectively, were very similar for the two conditions. Leaf sampling was performed at 20.8 d_20 °C_ for NS and 18.4 d_20 °C_ for ES (Online Resource 5 Fig. A). The mean ± SD (n = 30 genotypes) number of visible leaves per plant was 7.10 ± 0.40 for NS and 6.76 ± 0.30 for ES. This corresponds to about four ligulated leaves per plant (Table 2). However, for the 30 genotypes the vegetative plant growth was affected by the field condition as the dry weight of the seedling aerial part was significantly reduced by chilling induced by ES (Table 2, Wilcoxon rank test, *P* < 0.05). When, at this stage, biomasses were corrected for the thermal time in each condition (i.e. g DW divided by d_20 °C_ from emergence) the NS value (65 mg DW d_20 °C_^−1^) remained about twice the ES value (31 mg DW d_20 °C_^−1^), confirming a condition effect on growth. However, the final plant aerial biomass or grain biomass of mature plants at half milk line stage were not significantly affected by the sowing condition (*P* > 0.05, Table 2), showing that over their entire plant development period the ES plants performed equally. This compensation was mainly due to the longer cycle duration between emergence and harvest, and consequently the higher amount of cumulated thermal time (+ 10%) and incident global radiation (+ 21%) (Online Resource 5).

**Table 2:** Effect of sowing date on maize plant phenotype in the field at the time of leaf harvest for metabolite determination and on mature plants at the time of grain harvest

	Normal sowing	Early sowing
Young plants		
Number of ligulated leaves plant^−1^	4.51 ± 0.29	4.05 ± 0.10*
Total aerial vegetative biomass (g DW plant^−1^)	1.67 ± 0.48	0.71 ± 0.13*
Mature plants		
Final plant aerial biomass (g DW plant^−1^)	222.3 ± 28.0	230.6 ± 26.0
Final grain biomass (g DW plant^−1^)	108.3 ± 13.4	114.2 ± 12.9

Mean of 30 hybrid genotypes ± SD, except for NS total aerial vegetative biomass with 29 genotypes

*Statistically significant difference (*P* < 0.05) between the two conditions according to Wilcoxon rank test

### Leaf metabolite composition in the field is affected by early sowing

For ^1^H-NMR profiles, an a priori annotation work according to the literature resulted in the identification and absolute quantification of 24 compounds including five soluble sugars and one sugar-alcohol, seven organic acids, five amino acids and four other N-compounds (Online Resource 3). Five unknown compounds were also quantified. The major polar compounds were sucrose, *trans*-aconitate, malate, quinate, and glycine. For LC-ESI-QTOF-MS profiles, an annotation work performed at the beginning of the present study resulted in the identification and relative quantification of 25 compounds in the negative mode, including one amino acid, six hydroxycinnamate derivatives, 13 flavonoid derivatives, five benzoxazinoid derivatives (Online Resource 4). The three annotated compounds with the highest intensities were rutine, feruloylquinic acid and coumaroylquinic acid A. Maysin presented the highest intensity; however, it could not be quantified in our conditions due to signal saturation.

**Fig. 1:**
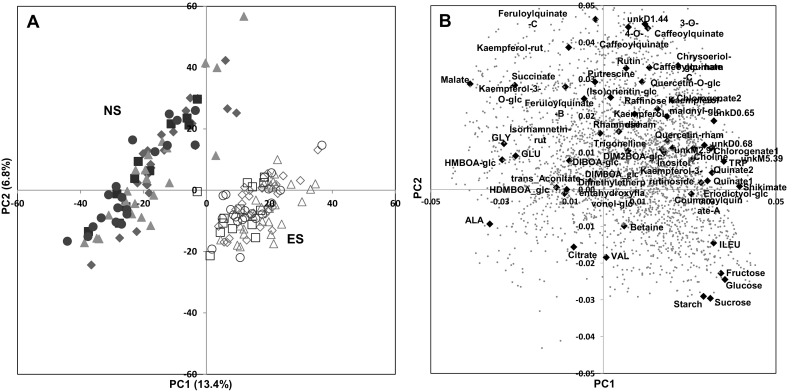
Principal component analysis of 2868 metabolite features and starch measured in young maize leaf of 30 hybrids in the normal (full symbols) and early-sowing (open symbols) conditions. **a** Scores plot of the first two principal components. Scores symbols correspond to the genotype groups defined in Table 1: European, green triangles; Iodent, purple circles; Lancaster, red squares; Stiff Stalk, blue diamonds; **b** Loadings plot of the first two principal components. Identified metabolites are annotated

To visualize leaf compositional changes, we performed a PCA for the two sowing conditions combining the compositional data from the three analytical approaches. The PCA scores plot (Fig. 1a) revealed that leaf composition clearly differed between the two conditions. The percentage of total variability explained by PC1 and PC2 was about 20%. On the PC1 × PC2 plane, the global compositional changes between genotypes were of the same order of magnitude as those within genotypes for a given sowing condition, and much lower than those induced by sowing date. No sample clustering linked with admixture groups (Fig. 1a) or silage-earliness groups (Online Resource 6) appeared. Comparison of the scores and loadings plots (Fig. 1b) highlighted the identified metabolites with higher contents for ES (glucose, fructose, sucrose, starch, isoleucine), or higher contents for NS (malate, succinate, glycine). A number of unidentified metabolite signatures from the LC-QTOF-MS also contributed to discriminate the two conditions, including 41 variables with values of PC1 loadings superior to 0.02 and PC2 loadings inferior to − 0.02, i.e. with a tendency to have higher contents in ES, and 77 variables with values of PC1 loadings inferior to − 0.02 and PC2 loadings superior to 0.02, i.e. with a tendency to have higher contents in NS. We used univariate analyses to verify the tendencies observed for the metabolites identified at the beginning of the present study, and also point to unidentified metabolites from the ^1^H-NMR or LC-QTOF-MS analyses that are the most significant and for which tentative identification would be particularly interesting.

**Fig. 2:**
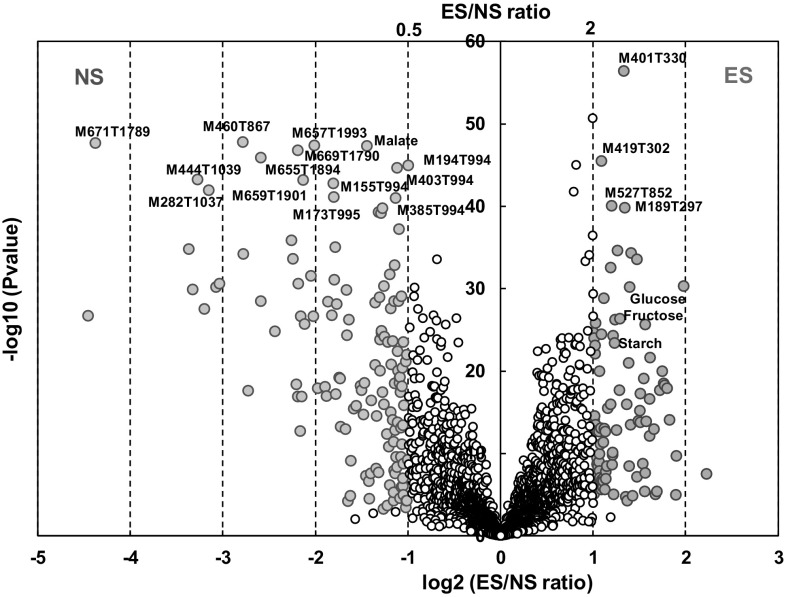
Volcano plot with Student’s *t* test for field sowing condition for all metabolite features and starch measured in young leaf of 30 maize hybrids cultivated in the field. The small and open dots correspond to variables with *P* > 0.001 or 0.5 < ES/NS ratio < 2. All MS-signatures with *P* < 10^−40^ and ES/NS ratio < 0.5 or > 2 are annotated. All identified compounds with *P* < 10^−20^ and ES/NS ratio < 0.5 or > 2 are annotated. Details with all variable names, ratios and *P*-values are presented in Online Resource 7 Tables

The results of Student’s *t* test (*P* < 0.01 or *P* < 0.001 after FDR correction) for sowing date effect for all variables, combined with a ratio of ES over NS condition higher than 1.2 or 2 or lower than 0.83 or 0.5, are detailed in Online Resource 7 tables, and summarized in Fig. 2 for the most stringent thresholds. All these compounds can be considered as metabolites responding to sowing date that are common to the majority of genotypes. The *P* < 0.01 and 1.2 or 0.83 ratio thresholds were used to point to significantly affected identified compounds. The *P* < 0.001 and 2 or 0.51 ratio thresholds were used to be more stringent for metabolite signatures to make a pre-selection before a tentative annotation work. The first set of thresholds allowed pointing to 25 identified compounds (Online Resource 7 Table A). Starch and two soluble sugars (glucose, fructose), two organic acids (quinate, shikimate), three amino acids or amino-compounds (isoleucine, choline, tryptophan), three phenolic compounds (kaempferol-malonyl-glucoside, coumaroylquinic-acid A, eriodictyol-*O*-glucoside), one benzoxazinoid (DIM_2_BOA-glucoside) had higher contents for ES. Three organic acids (malate, succinate, *trans*-aconitate), three amino acids or amino-compounds (putrescine, alanine, glutamate), four phenolic compounds (feruloylquinate C, two kaempferol derivatives and another flavonol-glycoside) and three benzoxazinoids (HDMBOA-glucoside, DIBOA-glucoside, HMBOA-glucoside) had higher contents for NS. These changes were summarized on a simplified metabolic map (Online Resource 8) including a benzoxazinoid pathway in agreement with the one proposed previously (Dutartre et al. 2012; Handrick et al. 2016). The second set of thresholds allowed pointing to 232 out of the 2869 variables (Online Resource 7 Table B, Fig. 2) including, among the identified compounds already noticed with *P* < 0.01, starch, glucose, fructose, and tryptophan that remained with higher contents for ES, and malate and HDMBOA-glucoside with higher contents for NS. Besides these identified compounds, 93 MS-based signatures had significantly higher contents in ES, and 134 MS-based signatures had significantly higher contents in NS. Although this corresponds to a response common to all genotypes, we checked that most of the 25 metabolites and 227 signatures highlighted above were also affected by genotype (ANOVAs, *P* < 0.05) in each sowing condition separately, which was the case for more than 80% of them. Among these 227 signatures, we pre-selected 40 of them with a *P* value < 10^−30^ for tentative annotation. After verification of signal intensity, formula assignment using precise mass and MS^2^ experiments, putative annotations of seven signatures were obtained (Online Resource 9). Among them, M707T993 was assigned to the [2M-H]^−^ dimer of caffeoylisocitrate, M713T795 to the [2M-H]^−^ dimer of the HMBOA-glucoside, M329T2126 to the [M-H]^−^ of tricin, and M224T977 to a fragment ion of HDMBOA-glucoside, and all four features presented significantly lower contents in the ES condition. M447T614 was assigned to the [M-H]^−^ of cyanidin-glucoside and M465T628 to the [M-H]^−^ of dihydroquercetin-glucoside and M675T639 to the [2M-H]^−^ of coumaroylquinate B, and all three features presented significantly higher contents in the ES condition.

### The variability of distances between genotypes based on leaf composition was lower under ES compared to NS condition

**Fig. 3:**
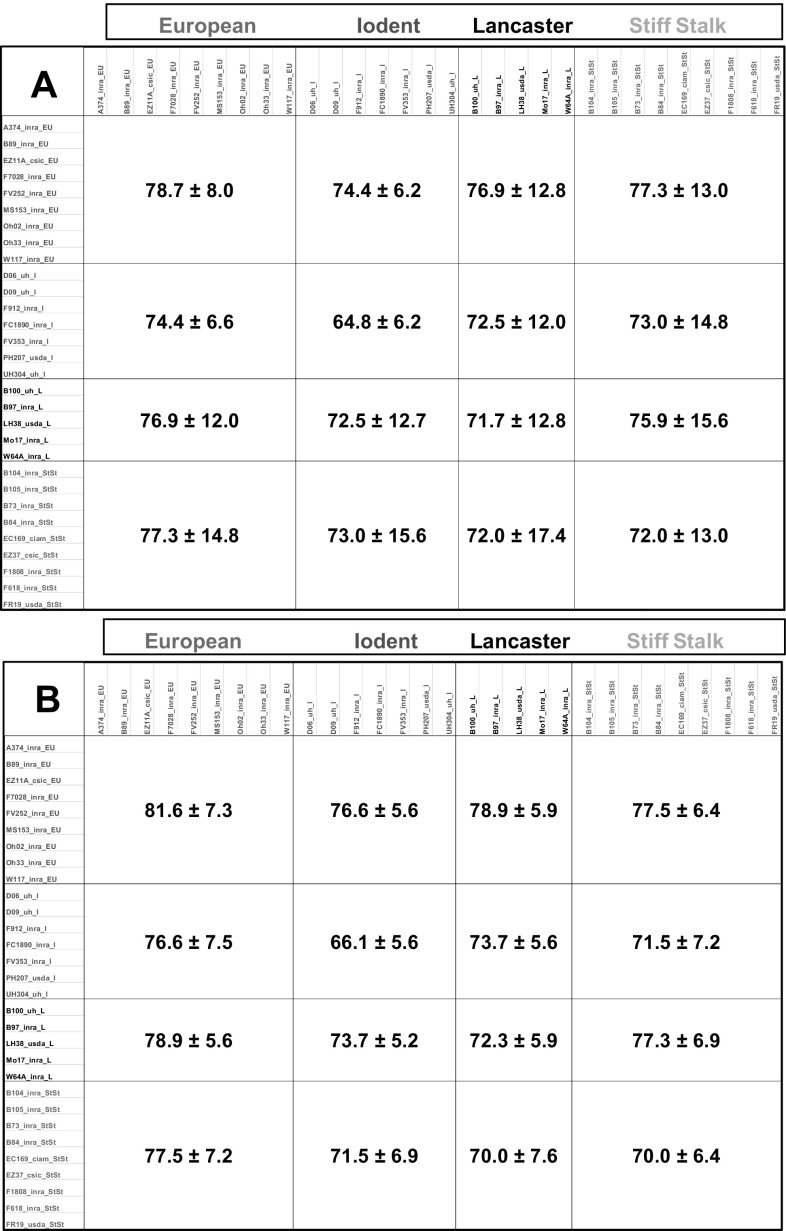
Euclidian distances between maize genotypes based on leaf compositional data. Mean ± standard deviation for distances intra- and inter-genotype groups. **a** NS condition. **b** ES condition

Euclidian distances were calculated based on the ls-means per genotype for compositional data separately for NS and ES conditions. The mean of all pair-wise distances were similar for the two conditions: 74.6 for NS and 75.4 for ES. However, the variability of all pair-wise distances was higher for the NS compared to the ES condition, with a coefficient of variation of 17.4% for NS compared to 10.1% for ES. Overall, the means of distances within or between the four genotype groups were similar (Fig. 3) showing that some compositional variability remains within each admixture group.

### A few metabolites or metabolite signatures seem to be linked with silage-earliness

**Fig. 4:**
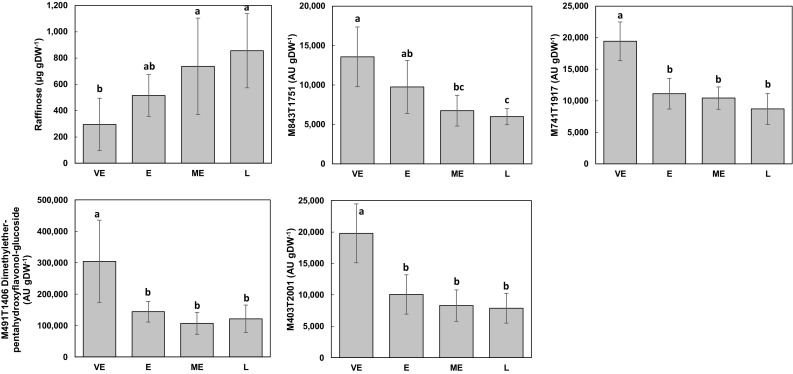
Mean contents for each silage-earliness group for the metabolites or metabolite features highlighted using VIP scores of OSC-OSC-PLS-DA of NS compositional data. *VE* very-early, *E* early, *ME* mid-early, *L* late, *AU* arbitrary units. Vertical bars represent standard deviations. For each variable, bars accompanied by the same letter are not significantly different according to Tukey’s test (*P* < 0.05)

Several silage-earliness groups were represented within each admixture group (Table 1). As the variability of all pair-wise distances was higher for the NS condition, we used this condition to search for metabolites linked with silage-earliness. We searched for possible links between leaf composition and genotype silage-earliness groups using OSC-PLS-DA on genotype ls-means. As only two genotypes were classified as “very late” in Table 1, they were gathered with “late” genotypes resulting in a total of 4 groups (very-early, early, mid-early, late). An OSC-OSC-PLS-DA with two components allowed separating the four silage-earliness groups along the first component as expected, with R^2^ of 0.045 and Q^2^ of 0.718. Twenty-five variables had a VIP score higher than 2.5. The mean values of variables with variable importance in the projection (VIP) scores for the first component higher than 2.5 for known compounds (raffinose and dimethyletherpentahydroxyflavonol-glucoside), and higher than 3 for unidentified metabolite signatures (M403T2001, M741T1917, M843T1751, Online Resource 9) are presented in Fig. 4. M403T2001, dimethyletherpentahydroxyflavonol-glucoside and M741T1917 presented significantly higher mean contents in the very-early group compared to all other earliness groups (Tukey’s test, *P* < 0.05). Mean M843T1751 content seemed to decrease from very-early to mid-early group. Tentative annotation of these three signatures did not succeed due to absence of candidates in MS databases and lack of structural information in MS spectra. Mean raffinose content seemed to increase linearly from very-early to late groups with significantly lower mean contents in the very-early group compared to mid-early or late silage-earliness groups. This behaviour was confirmed in a following year for raffinose absolute content in data obtained on the same genotypes and for NS (Online Resource 10). For the relative contents of MS-based variables, we used correlations between years for each variable to show that the genotype tendencies were confirmed for 15 of the 17 MS-based variables that matched one of the 24 MS-based variables of 2013 having a VIP score higher than 2.5 (Online Resource 10).

### Relationships between leaf metabolites and aerial biomass differ at the two sowing dates

**Fig. 5:**
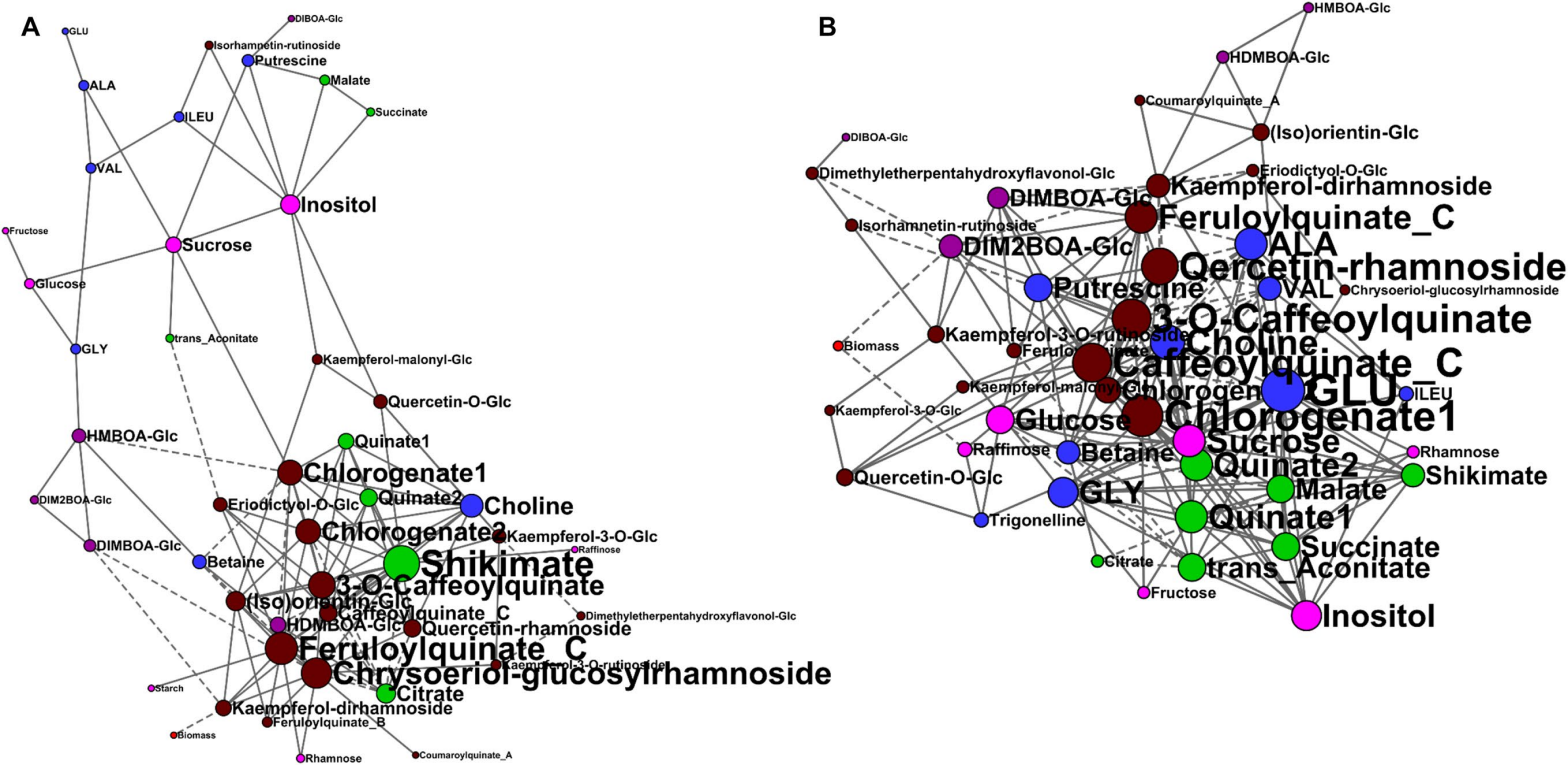
Networks of identified compounds and aerial biomass, based on correlations. Pearson correlations (after log2 transformation) in young leaf of maize hybrids cultivated in the field calculated with FDR correction (*q* < 0.02) and visualized using Cytoscape. Node size is proportional to the number of connections. Compound nodes are coloured according to the compound family: pink, sugars or sugar alcohols; green, organic acids; blue, amino acids and other amino compounds; purple, benzoxazinoids; brown, phenolic compounds. Biomass is coloured in red. Solid vertices, positive correlations; dashed vertices, negative correlations. **a** NS condition, 29 hybrids. **b** ES condition, 30 hybrids

As the plant aerial biomass has been measured a few days after leaf sample harvest, it became possible to study the relationship between biomass content and composition. Due to the data matrix dimensions, an approach based on multilinear regression with cross-validation did not appear feasible due to system undetermination. However, to visualize the relationship, correlation networks of compounds identified at the beginning of this study with aerial biomass were used using all available replicates (Fig. 5). Overall, the NS network (Fig. 5a) had lower density and average number of neighbors than the ES one (Fig. 5b): 0.11 and 5.0 versus 0.15 and 7.2 respectively. The larger network reconstructed for the NS condition (Fig. 5a) comprised 46 variables, out of the total of 48 variables, linked with 21 negative correlations and 98 positive correlations. The variables with at least 12 links were a caffeoylquinate, a feruloylquinate, chrysoeriol-glucosylrhamnoside and shikimate. The larger network reconstructed for the ES condition (Fig. 5b) comprised 46 variables linked with 33 negative correlations and 133 positive correlations. The variables with at least 12 links were several caffeoylquinates, a feruloylquinate as for NS, and also sucrose, quinate, alanine, glutamate, choline and a quercetin-rhamnoside. With the significance threshold chosen, starch was included in the NS network, but not in the ES one. Biomass was directly linked with kaempferol-dirhamnoside only for the NS condition network, whereas it was linked with DIM_2_BOA-glucoside and raffinose in the ES network, all with a negative correlation. Biplots for ls-means of the hybrids showed that each link between a metabolite content and aerial biomass does not seem to be due to a particular genotype admixture group (Online Resource 11).

## Discussion

The major compounds detected and quantified in the leaf of 30 hybrids grown in the field are globally in agreement with the literature for primary (Cañas et al. 2017; Richter et al. 2015; Sun et al. 2016b) and specialized metabolism (Korte et al. 2015; Meihls et al. 2013). Plant phenotypes and leaf metabolite modifications induced by sowing condition showed tendencies shared by the 30 genotypes and revealed metabolic responses. The compositional variability of the genotype panel was also used to estimate metabolic distances between genotypes, and highlight relationships with silage-earliness or plant aerial-biomass data.

### Metabolic response to chilling involves both primary and specialized metabolism

The environment exerted a larger effect on the leaf metabolome (Online Resource 8) than the genotype, in agreement with previous publications about cereals including maize (Baniasadi et al. 2014). For leaf primary metabolites, the balance between sugars and organic acids seemed to be modified by chilling with higher contents for ES in several carbohydrates (glucose, fructose, sucrose, starch), and lower contents in organic acids (malate, succinate), compared to NS. Such higher backup of carbohydrates suggests that photosynthetically fixed carbon was less efficiently used or accumulated under ES, and that growth was decreased whereas photosynthesis seemed less affected.

Alanine, produced in bundle sheath cells and with a high content in phloem sap (Valle and Heldt 1991), showed at least 12 connections in the ES correlation network and its content decreased in the ES condition. It will be interesting to evaluate the expression and the activity of alanine aminotransferase in these conditions, as this enzyme, which represents the primary route for the production of alanine, has been shown to enhance maize performance under N-limiting conditions when overexpressed (McAllister et al. 2012). Tryptophan, shikimic acid and quinic acid all had higher contents for ES. This may be linked with the reduction of growth and therefore a reduction in the lignification process, and/or a reduction of the synthesis of phenolic specialized metabolites due to a limitation of carbon availability as carbon preferentially accumulates in carbohydrates. Betaine, implicated in drought or chilling stress responses in several species, was not significantly increased in ES but its precursor choline was. Maize transformants accumulating significantly higher levels of betaine than wild-type plants have been shown to display less inhibition in seedling shoot growth rate (Quan et al. 2004).

For specialized metabolites such as hydroxycinnamates, flavonoids or benzoxazinoids, the responses to ES were finely tuned as they did not seem to concern entire pathway branches. Several caffeoylquinates or feruloylquinates showed at least 12 connections in the two networks and their contents did not seem to be modified by sowing date. Their homeostasis might be linked with their complex “house-keeping” roles for protection and the lignification process. However, two coumaroylquinic acid isomers, eriodyctiol-glucoside, a kaempferol-malonyl-glucoside, dihydroxyquercetin-glucoside and cyanidin-glucoside increased under ES. Several flavonols and anthocyanins have also been shown to accumulate upon cold exposure in Arabidopsis rosettes (Schulz et al. 2015). Similarly, for benzoxazinoids, a change in the balance from HDMBOA-glucoside to DIM_2_BOA-glucoside seemed to occur for ES. In addition to DIBOA-glucoside putatively identified at the beginning of our study, our complementary annotation work highlighted a HMBOA-glucoside which decreased in ES. Benzoxazinoids are constitutive or induced defence products against several biotic stresses (Handrick et al. 2016) that may provide a reliable signal for the induction of other defences (Meihls et al. 2013), including those against an abiotic stress (Bergvinson et al. 1995).

A range of other MS-based metabolite signatures also increased or decreased in response to ES. Besides the HMBOA-hexoside mentioned above, our tentative annotation work highlighted a caffeoylisocitrate that decreased under ES. The latter metabolite has been shown to be an abundant phenolic in the aerial part of orchard grass at vegetative stage (Hauck et al. 2013). To our knowledge, a possible role of this antioxidant compound in abiotic stress response has not been mentioned. Our tentative annotation work after the statistical analysis of the metabolomic profiles also highlighted a flavonoid, tricin, that decreased under ES. Tricin found in several grass species, including maize, is a monomer in grass lignification but free tricin seems to have other roles for adaptation to environment (Lan et al. 2016; Li et al. 2016) and was shown to decrease during cold acclimation in winter wheat leaves (Moheb et al. 2013).

Therefore, the ES condition induced the accumulation of several biomass precursors, compatible solutes and antioxidant compounds. Our metabolome results are in partial agreement with a recent proteomic study of a maize inbred-line cultivated in control and chilling condition showing that the adaptive response of maize seedlings to chilling stress includes alleviation of photodamage, more energy produced through glycolysis, and improvement in the overall ability to scavenge ROS (Wang et al. 2016).

### Metabolic plasticity seems to be constrained by chilling

No link appeared between leaf compositional characteristics and the genotype admixture groups. When leaf compositional data were used to calculate distances between hybrids, no direct link appeared between metabolic distances and the group of origin of the maternal lines of the hybrids based on Rincent et al. (2014). As the choice of the Dent panel representatives relied on their diversity based upon pedigree, genotyping and flowering dates, some diversity remained within a given group. Based on this diversity within and between groups, we revealed common responses of all genotypes as discussed above. However, the variability of all pair-wise distances between genotypes was lower for the ES compared to the NS condition.

The PCA scores plot, the metabolic distances within and between genotypes and the correlation networks (higher density and average number of neighbours for ES) all show that although ES did induce metabolic changes due to coordinated reprogramming, overall the metabolic plasticity between genotypes was lower under ES compared to NS. This may result from a sort of increased struggle for the allocation of resources to different competing needs (growth or defence against cold stress) under ES, creating possible trade-offs (Caretto et al. 2015). As the variability of all pair-wise distances between genotypes was higher for NS compared to ES, it may prove interesting to perform metabolomic phenotyping in control conditions especially in a breeding schema aiming at increasing a constitutive tolerance to a given stress. As a starting point, we looked at the link between leaf composition in NS condition and silage-earliness.

### Relationships between silage-earliness or aerial biomass and metabolite composition opens the way for new breeding strategies

The application of metabolomics to predict agronomic important phenotypes is emerging. Melchinger’s group in collaboration with MPIMPP Golm (Riedelsheimer et al. 2012a, b) compared the predictive power of metabolic and molecular markers and concluded that metabolites provide condensed information and could be especially interesting when dealing with highly polygenic traits. Their second study revealed significant correlations between caffeic- or *p*-coumaric acids and dry matter yield. A study on tropical maize hybrids revealed significant correlation between levels of glycine and *myo*-inositol and grain yield under drought (Obata et al. 2015). A recent study involving 19 maize lines (Cañas et al. 2017) defined a maize ideotype with a high grain yield potential with low accumulation of free amino acids and soluble carbohydrates in leaf. The latter study also proposed chlorogenates as markers that could be used to select for maize lines producing larger kernels.

In the present study, for two harvest years, we found a relationship between raffinose content and silage-earliness with very-early hybrids having the lowest raffinose content. Raffinose, often considered as a stress metabolite because it accumulates in plants experiencing abiotic stress, can be relatively abundant in stressed maize leaves. In a study comparing maize hybrids varying in drought tolerance, the drought-tolerant genotypes possessed a dwarf phenotype and accumulated more raffinose under milder drought-stress conditions than the intermediate or drought-susceptible genotypes (Barnaby et al. 2013). Raffinose may contribute to stabilize membranes, scavenge hydroxyl radicals and act as a signalling molecule during biotic stress (Van den Ende 2013). However, one may hypothesize that early hybrids invest less resource in raffinose, thus saving carbon and energy for growth. The negative correlation observed between aerial-biomass and raffinose leaf content under ES seems in line with this point.

Very-early hybrids also presented the highest content in the flavonol glucoside dimethyletherpentahydroxyflavonol-glucoside in 2013. In the present study, under NS, biomass was directly correlated to the content of kaempferol dirhamnoside, another flavonol glycoside, whereas it was correlated with DIM_2_BOA-glucoside under ES, all negatively. In Arabidopsis shoots, a compound similar to kaempferol dirhamnoside has been shown to act as an endogenous inhibitor of polar auxin transport (Yin et al. 2014). In the latter study, the loss of the flavonoid 3-*O*-glucosyltransferase UGT78D2 resulted in an altered flavonol glycoside pattern and reduced polar auxin transport in shoots, which was accompanied by reduced plant height and increased branching. In ES, the DIM_2_BOA-glucoside leaf content was higher than in NS condition, and became negatively linked with aerial biomass. This may be an indirect indication of a higher biotic stress level in ES, or rather an implication of benzoxazinoids in a “non-specific” stress response as indicated above. Indeed, in an early work about benzoxazinoids in maize shoots, 6-methoxy-2-benzoxazolinone (MBOA) has been described as a potent antiauxin (Hasegawa et al. 1992).

The fact that metabolites associated with biomass differed between the two growth conditions confirms that the use of metabolic markers for breeding for silage will necessitate precise and reproducible growth scenarios (Fernandez et al. 2016). Thus, validation of the potential markers proposed here for biomass (kaempferol-dirhamnoside for NS, DIM_2_BOA-glucoside and raffinose for ES condition) is needed for at least another year and with a larger genotype panel to confirm the present results. The validation of raffinose as a potential marker of silage-earliness for a second year with the present genotype panel is promising, as well as the correlations observed over 2 years for 15 MS-based variables implicated in the separation of silage-earliness genotype groups in 2013. Classical multiple regression modelling or other statistical methods such as those using supervised learning could be used (Broadhurst and Kell 2006). If confirmed, this suggests the possibility to develop low-cost analytical strategies targeting these “marker” metabolites to screen thousands of maize hybrids for breeding purposes (Fernandez et al. 2016). Raffinose could easily be analysed in high-throughput and at low costs using existing commercial kits and microplate robotized measurements (Gibon et al. 2012). Kaempferol-dirhamnoside or DIM_2_BOA-glucoside could be analysed using a targeted approach with a dedicated multiple reaction monitoring method using liquid chromatography coupled with tandem MS (Mwendwa et al. 2016; Engström et al. 2015). Several of the other MS-based potential markers still need to be identified to facilitate their high-throughput absolute quantification.

## Conclusions

The present study relies on 30 maize genotypes covering a range of genetic diversity. The metabolomic characterization of leaves of the maize hybrids cultivated in the field revealed chilling responses common to all these genotypes, and paved the way to search for individual metabolite markers or sets of metabolite markers of silage-earliness or aerial-biomass. Several metabolite signatures of interest remain to be characterized. When identified, their targeted measurements with higher throughput analytical methods may become possible. All the potential metabolite markers of genotype groups or global responses will have to be measured on larger genotype panels for validation before possible use for breeding purposes.

## Electronic supplementary material

Below is the link to the electronic supplementary material.

Online Resource 1 Environmental variables for the field experiment. Supplementary material 1 (PDF 350 KB)

Online Resource 2 Text detailing the ^1^H-NMR and LC-MS methods used for metabolomic profiling of maize leaves. Supplementary material 2 (PDF 299 KB)

Online Resource 3 Table of chemical shifts used for identification and quantification of metabolites in ^1^H-NMR spectra of polar extracts of maize leaf. Supplementary material 3 (PDF 462 KB)

Online Resource 4 List of metabolites putatively identified in semi-polar extracts of maize leaf by MS analysis. Supplementary material 4 (PDF 570 KB)

Online Resource 5 Figures for environmental conditions for the maize plants cultivated in a field: cumulative thermal times and cumulated incident photosynthetically active radiation. Supplementary material 5 (PDF 464 KB)

Online Resource 6 Figure of PCA of metabolite features and starch measured in young maize leaf of 30 hybrids in the NS and ES conditions. Scores plot as in Figure 1 annotated with genotype silage-earliness groups. Supplementary material 6 (PDF 415 KB)

Online Resource 7 Table of results of Volcano plot analysis (Fig. 2) for sowing condition for all metabolites or metabolite features and starch measured in leaf of maize hybrids. Supplementary material 7 (PDF 29 KB)

Online Resource 8 Changes in relative or absolute concentrations of identified or putatively-identified metabolites in maize leaf in response to ES summarized on metabolic maps. Supplementary material 8 (XLSX 166 KB)

Online Resource 9 Table for tentative annotation of several MS-based markers. Supplementary material 9 (PDF 134 KB)

Online Resource 10 Tentative validation of silage-earliness markers in 2014. Supplementary material 10 (PDF 308 KB)

Online Resource 11 Biplots of aerial biomass with the content of each metabolite directly linked with it in Figures 4A-B. Supplementary material 11 (PDF 428 KB)

## Abbreviations

d_20 °C_: Equivalent days at 20 °C after emergence
ES: Early sowing
LC-ESI-QTOF-MS: Liquid chromatography electrospray-ionization time-of-flight mass spectrometry
ls-means: Least-squares means
NS: Normal sowing
OSC-PLS-DA: Orthogonal signal correction partial least-square discriminant analysis
PCA: Principal component analysis
