# Supplementary material for: Metabotyping of 30 maize hybrids under early-sowing conditions reveals potential marker-metabolites for breeding
Source: Metabolomics. 2018 Sep 26;14(10):132. doi: 10.1007/s11306-018-1427-8 (PMC6208756; doi:10.1007/s11306-018-1427-8)
Supplement: Supplementary file 1 — Environmental variables for the field experiment. Supplementary material 1 (PDF 350 KB) [file 11306_2018_1427_MOESM1_ESM.pdf]

Journal: Metabolomics

Title: **Metabotyping of 30 maize hybrids under early-sowing conditions reveals potential marker metabolites for breeding**

Authors: Nadia Lamari, Vanessa Zhendre, Maria Urrutia, Stéphane Bernillon, Mickaël Maucourt, Catherine Deborde, Duyen Prodhomme, Daniel Jacob, Patricia Ballias, Dominique Rolin, Hélène Sellier, Dominique Rabier, Yves Gibon, Catherine Giauffrey, Annick Moing

Supplementary Table S1: Environmental variables for the field experiment for two following plant development and growth periods for each sowing condition: sowing to emergence, and emergence to leaf sample harvest. ES, early-sowing. NS, normal-sowing.

**Sowing condition**	**Growth period**	**Duration of period** (days)	**Mean global radiation** (J/cm² per day)	**Total rainfall over the period** (mm)	**Mean air temperature** (°C)	**Maximum air vapor pressure deficit** (Pa)
ES	Sowing to emergence	19	1 727	10.0	10.8	1.99
	Emergence to leaf sample harvest	41	1 620	90.5	12.1	1.85
NS	Sowing to emergence	22	1 467	61.5	10.4	1.22
	Emergence to leaf sample harvest	31	1 654	78.0	15.9	1.85
