# Supplementary material for: Metabotyping of 30 maize hybrids under early-sowing conditions reveals potential marker-metabolites for breeding
Source: Metabolomics. 2018 Sep 26;14(10):132. doi: 10.1007/s11306-018-1427-8 (PMC6208756; doi:10.1007/s11306-018-1427-8)
Supplement: Supplementary file 2 — Text detailing the 1H-NMR and LC-MS methods used for metabolomic profiling of maize leaves. Supplementary material 2 (PDF 299 KB) [file 11306_2018_1427_MOESM2_ESM.pdf]

Journal: Metabolomics

Title: **Metabotyping of 30 maize hybrids under early-sowing conditions reveals potential marker metabolites for breeding**

Authors: Nadia Lamari, Vanessa Zhendre, Maria Urrutia, Stéphane Bernillon, Mickaël Maucourt, Catherine Deborde, Duyen Prodhomme, Daniel Jacob, Patricia Ballias, Dominique Rolin, Hélène Sellier, Dominique Rabier, Yves Gibon, Catherine Giauffrey, Annick Moing

Supplementary text 1.

Detailed ^{1}H-NMR and LC-QTOF-MS methods for metabolomic profiling of maize leaves.

^{1}H-NMR analysis of polar metabolites

For NMR analysis of field samples, polar metabolites were extracted from 20 mg DW using a hot ethanol/water series and quantified by ^{1}H-NMR as previously described (Biais *et al.,* 2009) with special care to allow absolute quantification of individual metabolites. The pellets were kept for starch analysis. The lyophilized polar extracts were dissolved in 500 µl of deuterated phosphate buffer solution (200 mM, apparent pH 6.00), titrated with deuterared KOD 1 M or DCL 0.1 M solutions by means of BTpH (Bruker BioSpin GmbH, Karlsruhe, Germany) to pH 6.00 ± 0.02 when necessary, and lyophilized again. Each dried titrated extract was solubilized in 0.5 ml of D_2O with (trimethylsilyl) propionic-2,2,3,3-d4 acid (TSP) sodium salt (0.01% final concentration) for chemical shift calibration and EDTA.Na_2 (4 mM final concentration). Quantitative ^{1}H-NMR spectra were recorded at 500.162 MHz on a Bruker Avance III spectrometer (Bruker, Karlsruhe, Germany) using an ATMA-BBI 5 mm probe flushed with nitrogen gas and an electronic reference for quantification (ERETIC2). Sixty-four scans of 32k data points each were acquired with a 90 ° pulse angle, a 6000 Hz spectral width, a 2.73 s acquisition time, and a 25 s recycle delay. Preliminary data processing was conducted with TOPSPIN 3.2 software (Bruker). For absolute quantification of metabolites, three calibration curves (glucose and fructose: 1.25 to 50 mM, glutamate: 0 to 15 mM) were prepared and analysed under the same conditions. The glucose calibration was used for the absolute quantification of all compounds, as a function of the number of protons of selected resonances except fructose and glutamate quantified using their respective calibration curve. The metabolite concentrations were calculated using AMIX (version 3.9.14, Bruker) software.

LC-QTOF-MS analysis of semi-polar metabolites

Lyophilized maize samples (20 ± 0.5 mg DW) from the field experiment were extracted with 1 mL of methanol/water (70/30, v/v) with 0.1% formic acid and methyl vanillate as internal standard. After vortexing, the suspensions were sonicated for 15 min at a constant temperature of 0°C. The resulting

homogenates were centrifuged at 14,462 *g* for 5 min at room temperature and the supernatants were filtrated (0.22 µm PVDF filter, Merck Millipore, Darmstadt, Germany). Methanolic extracts were analyzed by LC-QTOF-MS. Chromatographic runs were conducted with a Dionex Ultimate 3000 system (Thermo Scientific, Bremen, Germany) using a reverse phase column (C_{18}-Gemini 2.0 x 150 mm, 3 µm, 110Å, Phenomenex, Torrance, CA, USA) with a flow rate of 350 µl/min. The mobile phase consisted in nanopure water with 0.1% formic acid (A) and acetonitrile (B) with the following elution gradient: 0-3 min, 5% B; 3-33 min, 5-30% B; 33-38 min, 30-95% B; 38-43 min, 95% B. Mass spectral analyses were performed with a hybrid quadrupole/time-of-flight mass spectrometer (micrOTOF-*Q*, Bruker Daltonics, Bremen, Germany) for the full scan mode acquisitions. The instrument was equipped with an ESI source in negative-ion mode (-500 V endplate offset, +3.5 kV capillary voltage, 2.4 bar nebulizer, dry gas flow of 8.0 L/min at 190° C) was operated in a 50-1500 *m/z* mass range. MS^2 experiments and injections of authentic commercial compounds (Sigma, Saint-Quentin Fallavier, France or Extrasynthèse, Genay, France) were performed for annotation purpose. Molecular formula were generated using SmartFormula software (Bruker, Bremen, Germany). An error of 3 mDa and mSigma of 50 were generally considered as acceptable upper limits.

The data was processed using Workflow4Metabolomics in the Galaxy environment (Giacomoni *et al.*, 2015) for i) pre-processing LC-MS data using XCMS functions, ii) filtration and normalization, iii) first statistical analyses such as ANOVA. A total of 7,307 chemical features were detected from HPLC-micrOTOF-*Q*-MS. Data reduction was achieved using four filtering steps: (1) removal of contaminant peaks found in blank extraction; (2) coefficient of variation (CV) ratio of samples over QC samples threshold of 1.25; (3) removal of non-useful chromatographic regions, such as dead volume and equilibration time, and the redundant isotopes, such as $[M+1]^-$, $[M+2]^-$, $[M+2]^{2-}$, $[M+1]^{2-}$ and $[M]^{2-}$; (4) two-factor ANOVA (P<0.05) (sowing date and genotype) with false discovery rate adjustment.

References

Biais B, Allwood JW, Deborde C, Xu Y, Maucourt M, Beauvoit B, Dunn WB, Jacob D, Goodacre R, Rolin D, Moing A. 2009. H-1 NMR, GC-EI-TOFMS, and data set correlation for fruit metabolomics: Application to spatial metabolite analysis in melon. *Analytical Chemistry* 81, 2884-2894.

Giacomoni F, Le Corguillé G, Monsoor M, Landi M, Pericard P, Pétéra M, Duperier C, Tremblay-Franco M, Martin J-F, Jacob D, Goulitquer S, Thévenot EA, Caron C. 2015. Workflow4Metabolomics: A collaborative research infrastructure for computational metabolomics. *Bioinformatics* 31, 1493-1495.
