# Supplementary material for: Metabotyping of 30 maize hybrids under early-sowing conditions reveals potential marker-metabolites for breeding
Source: Metabolomics. 2018 Sep 26;14(10):132. doi: 10.1007/s11306-018-1427-8 (PMC6208756; doi:10.1007/s11306-018-1427-8)
Supplement: Supplementary file 3 — Table of chemical shifts used for identification and quantification of metabolites in 1H-NMR spectra of polar extracts of maize leaf. Supplementary material 3 (PDF 462 KB) [file 11306_2018_1427_MOESM3_ESM.pdf]

Journal: Metabolomics

Title: **Metabotyping of 30 maize hybrids under early-sowing conditions reveals potential marker metabolites for breeding**

Authors: Nadia Lamari, Vanessa Zhendre, Maria Urrutia, Stéphane Bernillon, Mickaël Maucourt, Catherine Deborde, Duyen Prodhomme, Daniel Jacob, Patricia Ballias, Dominique Rolin, Hélène Sellier, Dominique Rabier, Yves Gibon, Catherine Giauffrey, Annick Moing

Supplementary Table S2: Table of chemical shifts used for identification and quantification of metabolites in ^{1}H-NMR spectra of polar extracts (in deuterated phosphate buffer solution, apparent pH 6.0) of maize leaf, expressed as relative values to the TSP resonance at 0 ppm. s: singlet, d: doublet, bd: broad doublet, dd: doublet of doublets, t: triplet, td: triplet of doublets, m: multiplet.

Compounds	Assignment	^{1}H Chemical shift (ppm)	^{1}H Pattern	Use (quantification or detection)	MSI status [a]
**Alanine**	β-CH_3	1.487	d	q	1
**Betaine**	N-CH_3	3.27	s	q	1
**Chlorogenate1 (hydroxycinnamic-containing compound)**	=CH	6.42	d	q	3
	CH_{arom}	6.93	d	d	
	CH_{arom}	7.12	dd	d	
	CH_{arom}	7.19	d	d	
	=CH	7.63	d	d	
**Chlorogenate2 (hydroxycinnamic and quinic-containing compound)**	=CH	6.36	d	q	2
	CH_{arom}	6.98	d	d	
	CH_{arom}	7.09	dd	d	
	CH_{arom}	7.16	d	d	
	=CH	7.62	d	d	
	quinic H5	5.32	m	d	
	quinic H?	4.25	m	d	
**Choline**	N-CH_3	3.2	s	q	1
	N-CH_2	3.53	m	d	
**Citrate**	CH_2	2.7	d	q	1
	CH_2	2.55	d	d	
**Fructose**		4.12	m	q	1
**Glucose**	α-CH	5.24	d	q	1
	β-CH	4.65	d	q	1
**Glutamate**	γ-CH_2	2.36	td	q	1
**Glycine**	α-CH	3.56	s	q	1
**Inositol**	C_5H	3.29	t	q	1
**Isoleucine**	δ-CH_3	0.94	t	d	1
	γ′-CH_3	1.01	d	q	
**Leucine**	δ-CH_3	0.96	d	d	1
	δ′-CH_3	0.97	d	d	1
**Malate**	α-CH	4.31	dd	q	1
	β-CH	2.68	dd	d	
	β′-CH	2.39	dd	d	
**Putrescine**	CH_2	1.77	m	q	1

Putrescine	N-CH_2	3.06	m	d	
**Quinate1**		1.93	dd	q	3
**Quinate2 (probably free quinic acid)**		1.88	dd	q	3
		1.96	m	d	
		1.98	m	d	
		2.04	d	d	
		2.07	m	d	
		4.03	m	d	
		4.15	dd	d	
**Raffinose**		4.99	d	q	2
**Rhamnose**		5.12	d	q	1
		1.3	d	d	
		1.28	d	d	
**Shikimate**		4.04	dd	q	1
		2.79	bd	d	
		2.75	bd	d	
**Succinate**	CH_2	2.42	s	q	1
**Sucrose**		5.42	d	q	1
		4.22	d	d	
		4.06	t	d	
		3.9	m	d	
		3.82	m	d	
		3.77	t	d	
		3.68	s	d	
		3.57	dd	d	
		3.48	t	d	
**Trans-aconitate**	=CH	6.62	s	q	1
	CH_2	3.47	s	d	
**Trigonelline**	=CH-N	9.12	s	q	1
	CH-C=C-N	8.84	m	d	
	CH=C-N	8.09	t	d	
	CH_3	4.45	s	d	
**Valine**	γ′-CH_3	0.99	d	q	1
**Unknown compounds**					
**UnkD0.65**		0.65	d	q	4
**UnkD0.68**		0.68	d	q	4
**UnkD1.44**		1.44	d	q	4
**UnkM2.91**		2.91	m	q	4
**UnkM5.39**		5.39	m	q	4

[a] Identification status according to MSI (Sumner *et al.* , 2007): 1, Identified compounds (checked with standard); 2, Putatively annotated compounds; 3, Putatively characterized compound classes; 4, Unknown.
