# Supplementary material for: Metabotyping of 30 maize hybrids under early-sowing conditions reveals potential marker-metabolites for breeding
Source: Metabolomics. 2018 Sep 26;14(10):132. doi: 10.1007/s11306-018-1427-8 (PMC6208756; doi:10.1007/s11306-018-1427-8)
Supplement: Supplementary file 4 — List of metabolites putatively identified in semi-polar extracts of maize leaf by MS analysis. Supplementary material 4 (PDF 570 KB) [file 11306_2018_1427_MOESM4_ESM.pdf]

Journal: Metabolomics

Title: **Metabotyping and chilling response of 30 maize hybrids** Authors: Nadia Lamari, Vanessa Zhendre, Maria Urrutia, Stéphane Bernillon, Mickaël Maucourt, Catherine Deborde, Duyen Prodhomme, Daniel Jacob, Patricia Ballias, Dominique Rolin, Hélène Sellier, Dominique Rabier, Yves Gibon, Catherine Giauffrey, Annick Moing

Supplementary Table S3: LC-QTOF-MS and MS/MS data of identified compounds in maize leaves in negative ion-mode.

RT (min)	Measured *m/z* of [M-H]$^-$	MS spectrum ions	Putative Molecular Formula of [M-H]$^-$	Calculated m/z of [M-H]$^-$	Error (mDa)	Error (ppm)	mSigma value	Putative Assignment	ChEBI ID	LC-MS/MS in negative mode [a]	Comparison with Spectral DB[b]	Comparison with chemical standard	MSI level[c]	Variable associated to [M-H]$^-$
4.78	203.0828	-	$C_{11}H_{11}N_2O_2$	203.0826	-0.2	-1.1	3.9	Tryptophan		Signal too low	-	yes	1	M203T290
6.75	342.0831	180.0297 [(M-H]-$C_6H_{10}O_5]^-$<br>685.1680 [2M-H]$^-$	$C_{14}H_{16}NO_9$	342.031	-0.1	-0.2	0.7	DIBOA-glucoside	CHEBI:80091	180.0304 [(M-H]-$C_6H_{10}O_5]^-$<br>162.0206 [(M-H]-$C_6H_{12}O_6]^-$<br>152.0351 [(M-H]-$C_7H_{10}O_6]^-$	-	no	3	M342T407
10.41	337.0921	163.0395 [(M-H]-$C_7H_{10}O_5]^-$<br>675.1817 [2M-H]$^-$	$C_{16}H_{17}O_8$	337.0929	0.8	2.4	2.6	Coumaroylquinic acid A	-	191.0559 [(M-H]-$C_9H_6O_2]^-$<br>173.0454 [(M-H]-$C_9H_8O_3]^-$<br>163.0397 [(M-H]-$C_7H_{10}O_5]^-$	-	no	2	M337T627
11.79	353.0861	191.0557 [(M-H]-$C_9H_6O_3]^-$<br>707.1702 [2M-H]$^-$	$C_{16}H_{17}O_9$	353.0978	1.7	4.9	3.2	3-O-Caffeoylquinic acid	CHEBI:16112	191.0565 [(M-H]-$C_9H_6O_3]^-$<br>179.0343 [(M-H]-$C_7H_{10}O_5]^-$<br>173.0451 [(M-H]- $C_9H_8O_4]^-$<br>161.0246 [(M-H]- $C_7H_{12}O_6]^-$	MoNA<br>mzCloud	yes	1	M353T707
12.39	367.1018	193.0499 [(M-H]-$C_7H_{10}O_5]^-$<br>735.1971 [2M-H]$^-$	$C_{17}H_{19}O_9$	367.1035	1.7	4.6	20.2	Feruloylquinic acid B	-	193.0507 [(M-H]-$C_7H_{10}O_5]^-$<br>191.0562 [(M-H]-$C_{10}H_8O_3]^-$<br>173.0459 [(M-H]-$C_{10}H_{10}O_4]^-$	-	no	2	M367T741
12.65	353.0864	173.0446 [(M-H]- $C_9H_8O_4]^-$<br>707.1705 [2M-H]$^-$	$C_{16}H_{17}O_9$	353.0878	1.2	3.5	29.7	4-O-Caffeoylquinic acid	CHEBI:75491	191.0563 [(M-H]-$C_9H_6O_3]^-$<br>179.0351 [(M-H]-$C_7H_{10}O_5]^-$<br>173.0456 [(M-H]- $C_9H_8O_4]^-$<br>161.0250 [(M-H]- $C_7H_{12}O_6]^-$	-	yes	1	M353T757
13.18	356.1008	194.0481 [(M-H]-$C_6H_{10}O_5]^-$<br>713.2035 [2M-H]$^-$	$C_{15}H_{18}NO_9$	356.0987	-2.1	-5.8	45.1	HMBOA-glucoside	CHEBI:134457	341.0738 [(M-H]-$CH_3^{\bullet}]^{\bullet -}$<br>194.0458 [(M-H]-$C_6H_{10}O_5]^-$<br>179.0221 [(M-H]-$C_6H_{10}O_5$ $CH_3^{\bullet}]^{\bullet -}$<br>166.0505 [v-$C_7H_{10}O_6]^-$	-	no	3	M356T796
13.40	372.0932	192.0300 [(M-H]-$C_6H_{12}O_6]^-$<br>210.0413 [(M-H]-$C_6H_{10}O_5]^-$<br>745.1920 [2M-H]$^-$	$C_{15}H_{18}NO_{10}$	372.0936	0.4	1.1	5.0	DIMBOA-glucoside	CHEBI:16603	210.0407 [(M-H]-$C_6H_{10}O_5]^-$<br>192.0274 [(M-H]-$C_6H_{12}O_6]^-$<br>189.0050 [(M-H]-$C_9H_{13}NO_3]^-$<br>164.0344[(M-H]-$C_7H_{12}O_7]^-$<br>149.0111 [(M-H]-$C_8H_{15}O_7^{\bullet}]^{\bullet -}$	-	no	3	M372T817
13.67	402.1019	805.1879 [2M-H]$^-$	$C_{16}H_{20}NO_{11}$	402.1042	2.3	5.8	6.2	DIM$_2$BOA-glucoside	-	194.0437 [(M-H]-$C_{10}H_{10}NO_4]^-$<br>179.0225 [(M-H]-$C_8H_{15}O_7]^-$<br>163.9996 [(M-H]-$C_9H_{18}O_7]^-$	-	no	3	M402T823
13.78	353.0871	191.0559 [(M-H]-$C_9H_6O_3]^-$<br>707.1800 [2M-H]$^-$	$C_{16}H_{17}O_9$	353.0878	0.7	2.1	4.3	Caffeoylquinic acid C	-	191.0562 [(M-H]-$C_9H_6O_3]^-$<br>179.0298 [(M-H]-$C_7H_{10}O_5]^-$<br>173.0448 [(M-H]- $C_9H_8O_4]^-$<br>161.0238 [(M-H]- $C_7H_{12}O_6]^-$	-	no	3	M353T832
15.67	533.0982	-	$C_{24}H_{21}O_{14}$	533.0937	4.5	8	52.7	Kaempferol-malonylglucoside		287.0775 [(M-H]-$C_9H_{11}O_8]^-$	-	no	3	M533T931
16.05	386.1084	224.0560 [(M-H]-$C_6H_{10}O_5]^-$	$C_{16}H_{20}NO_{10}$	386.1093	0.9	2.3	0.4	HDMBOA-glucoside	-	224.0563 [(M-H]-$C_6H_{10}O_5$]-<br>196.0620 [(M-H]-$C_7H_{10}O_6$]-<br>168.0665 [(M-H]-$C_8H_{10}O_7$]-	-	no	3	M386T977
16.66	367.1024	191.0542 [(M-H]-$C_{10}H_8O_3]^-$	$C_{17}H_{19}O_9$	367.1035	1.0	2.8	4.4	Feruloylquinic acid C	-	191.0558 [(M-H]-$C_{10}H_8O_3]^-$	-	no	3	M367T1003
18.60	593.1531	-	$C_{27}H_{29}O_{15}$	593.1512	-1.9	-3.2	8.7	(Iso)orientin-glucoside	-	473.01078 [(M-H]-$C_4H_8O_4]^-$<br>447.0938 [(M-H]-$C_6H_{10}O_4]^-$<br>429.0816[(M-H]-$C_6H_{12}O_5]^-$<br>357.0602 [(M-H]-$C_9H_{16}O_7]^-$<br>327.0497 [(M-H]-$C_{10}H_{18}O_8]^-$<br>285.0392 [(M-H]-$C_{12}H_{20}O_9]^-$	-	no	3	M593T1114
19.71	449.1074	287.0555 [(M-H]-$C_6H_{10}O_5]^-$	$C_{21}H_{21}O_{11}$	449.1089	1.5	3.3	5.1	Eriodictyol-glucoside	CHEBI:65860	287.0532 [(M-H]-$C_6H_{10}O_5]^-$<br>193.0159 [(M-H]-$C_{12}H_{16}O_6]^-$<br>151.0031 [(M-H]-$C_{14}H_{18}O_7]^-$	-	no	3	M449T1234

20.85	577.1556	-	$C_{27}H_{29}O_{14}$	577.1563	0.6	1.1	5.1	Kaempferol-dirhamnoside		458.1141 [(M-H]-$C_4H_8O_4$]$^-$<br>413.0863 [(M-H]-$C_6H_{12}O_5$]$^-$<br>293.0440 [(M-H]-$C_{10}H_{20}O_9$]$^-$	-	no	2	M577T1253
21.08	609.1481	-	$C_{27}H_{29}O_{16}$	609.1461	0.8	1.3	6.6	Rutin	CHEBI:28527	463.0881 [(M-H]-$C_6H_{10}O_4$]$^-$<br>300.0269 [(M-H]-$C_{12}H_{21}O_9^{\bullet}$]$^{\bullet-}$	mzCloud	yes	1	M609T1268
21.79	463.0880	927.1787 [2M-H]$^-$	$C_{21}H_{19}O_{12}$	463.0882	0.2	0.5	1.7	Quercetin-*O*-glucoside	CHEBI:64621	300.0273 [(M-H]-$C_6H_{12}O_5^{\bullet}$]$^{\bullet-}$<br>271.0236 [[(M-H]-$C_7H_{12}O_6$]$^-$	mzCloud	no	3	M463T1312
23.40	491.1173	-	$C_{23}H_{23}O_{12}$	491.1195	2.2	4.6	47.7	Dimethyletherpentahydrox yflavonol -glucoside	-	476.0903 [(M-H]-$CH_3^{\bullet}$]$^{\bullet-}$<br>371.0774 [(M-H]-$C_4H_8O_4$]$^-$<br>329.0642[(M-H]-$C_6H_{10}O_5$]$^-$<br>314.0412[(M-H]-$C_7H_{13}O_5^{\bullet}$]$^{\bullet-}$<br>299.0200[(M-H]-$C_8H_{16}O_5$]$^-$	-	no	3	M491T1406
23.47	593.1516	629.1279 [M+Cl]$^-$	$C_{27}H_{29}O_{15}$	593.1512	-0.4	-0.7	3.5	Kaempferol-3-*O*-rutinoside	CHEBI:69657	447.0900 [(M-H]-$C_6H_{10}O_4$]$^-$<br>285.0392 [(M-H]-$C_{12}H_{20}O_9$]$^-$	-	yes	1	M593T1412
24.00	623.1623	-	$C_{28}H_{31}O_{16}$	623.1618	-0.5	-0.8	4.0	Isorhamnetin-rutinoside	-	315.0493 [(M-H]-$C_{12}H_{20}O_9$]$^-$<br>300.0260 [(M-H]-$C_{13}H_{23}O_9^{\bullet}$]$^{\bullet-}$	-	no	3	M623T1442
24.27	447.0930	-	$C_{21}H_{19}O_{11}$	447.0933	0.3	0.6	9.8	Kaempferol-3-*O*-glucoside	CHEBI:30200	284.0317 [(M-H]-$C_6H_{11}O_6^{\bullet}$]$^{\bullet-}$<br>255.0292 [(M-H]-$C_7H_{12}O_6$]$^-$<br>227.0341 [(M-H]-$C_8H_{12}O_7$]$^-$	MoNA	no	2	M447T1460
25.09	447.0925	-	$C_{21}H_{19}O_{11}$	447.0933	0.8	1.7	2.0	Quercetin rhamnoside	-	357.0601 [(M-H]-$C_3H_6O_3$]$^-$<br>327.0501 [(M-H]-$C_4H_8O_4$]$^-$<br>298.0466 [(M-H]-$C_5H_9O_5^{\bullet}$]$^{\bullet-}$	-	no	3	M447T1510
26.89	575.1414	-	$C_{27}H_{27}O_{14}$	575.1406	-0.8	-1.4	4.4	Maysin	CHEBI:70206	473.1078 [(M-H]-$C_4H_6O_3$]$^-$<br>411.0709 [(M-H]-$C_6H_{12}O_5$]$^-$<br>297.0395 [(M-H]-$C_{11}H_{18}O_8$]$^-$	-	no	3	M575T1614
30.93	607.1662	-	$C_{28}H_{31}O_{15}$	607.1668	0.6	1.1	2.3	Chrysoeriol-glucosylrhamnoside		575.1403 [(M-H]-CH_4O]$^-$<br>473.1092 [(M-H]-$C_5H_{10}O_4$]$^-$<br>411.0708 [(M-H]-$C_7H_{16}O_6$]$^-$<br>297.0401 [(M-H]-$C_{12}H_{22}O_9$]$^-$	-	no	3	M607T1733

[a] Precursor was [M-H]$^-$. AutoMS^2 mode collision energies were set according to *m/z* ratios of precursor.

[b] MassBank.jp: http://www.massbank.jp/ ; MoNA: http://mona.fiehnlab.ucdavis.edu/ ; mzCloud: https://www.mzcloud.org/ .

[c] According to Sumner et al. 2007 (Metabolomics 3:211-221).
