# Supplementary material for: Metabotyping of 30 maize hybrids under early-sowing conditions reveals potential marker-metabolites for breeding
Source: Metabolomics. 2018 Sep 26;14(10):132. doi: 10.1007/s11306-018-1427-8 (PMC6208756; doi:10.1007/s11306-018-1427-8)
Supplement: Supplementary file 5 — Figures for environmental conditions for the maize plants cultivated in a field: cumulative thermal times and cumulated incident photosynthetically active radiation. Supplementary material 5 (PDF 464 KB) [file 11306_2018_1427_MOESM5_ESM.pdf]

Journal: Metabolomics

Title: **Metabotyping of 30 maize hybrids under early-sowing conditions reveals potential marker metabolites for breeding**

Authors: Nadia Lamari, Vanessa Zhendre, Maria Urrutia, Stéphane Bernillon, Mickaël Maucourt, Catherine Deborde, Duyen Prodhomme, Daniel Jacob, Patricia Ballias, Dominique Rolin, Hélène Sellier, Dominique Rabier, Yves Gibon, Catherine Giauffrey, Annick Moing

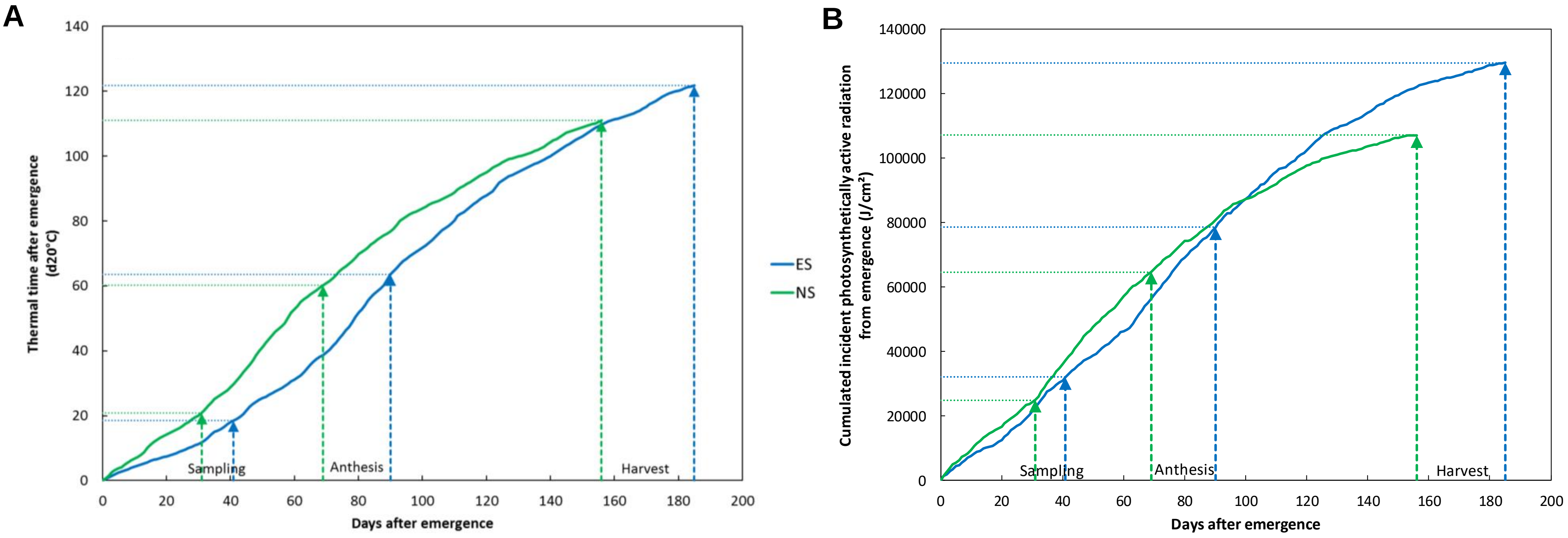

Supplementary Figure S1: Environmental conditions for the maize plants cultivated in a field from emergence to grain maturity. Blue, early sowing (ES); Green, normal sowing (NS). The three pairs of arrows indicate the time of leaf sampling for the biochemical analyses, plant anthesis and final harvest.

A. Cumulative thermal times. Thermal times were calculated as the equivalent number of days at 20°C after emergence for each sowing condition. B. Cumulated incident photosynthetically active radiation.
