# Supplementary material for: Metabotyping of 30 maize hybrids under early-sowing conditions reveals potential marker-metabolites for breeding
Source: Metabolomics. 2018 Sep 26;14(10):132. doi: 10.1007/s11306-018-1427-8 (PMC6208756; doi:10.1007/s11306-018-1427-8)
Supplement: Supplementary file 6 — Figure of PCA of metabolite features and starch measured in young maize leaf of 30 hybrids in the NS and ES conditions. Scores plot as in Figure 1 annotated with genotype silage-earliness groups. Supplementary material 6 (PDF 415 KB) [file 11306_2018_1427_MOESM6_ESM.pdf]

Journal: Metabolomics
Title: **Metabotyping of 30 maize hybrids under early-sowing conditions reveals potential marker metabolites for breeding**
Authors: Nadia Lamari, Vanessa Zhendre, Maria Urrutia, Stéphane Bernillon, Mickaël Maucourt, Catherine Deborde, Duyen Prodhomme, Daniel Jacob, Patricia Ballias, Dominique Rolin, Hélène Sellier, Dominique Rabier, Yves Gibon, Catherine Giauffrey, Annick Moing

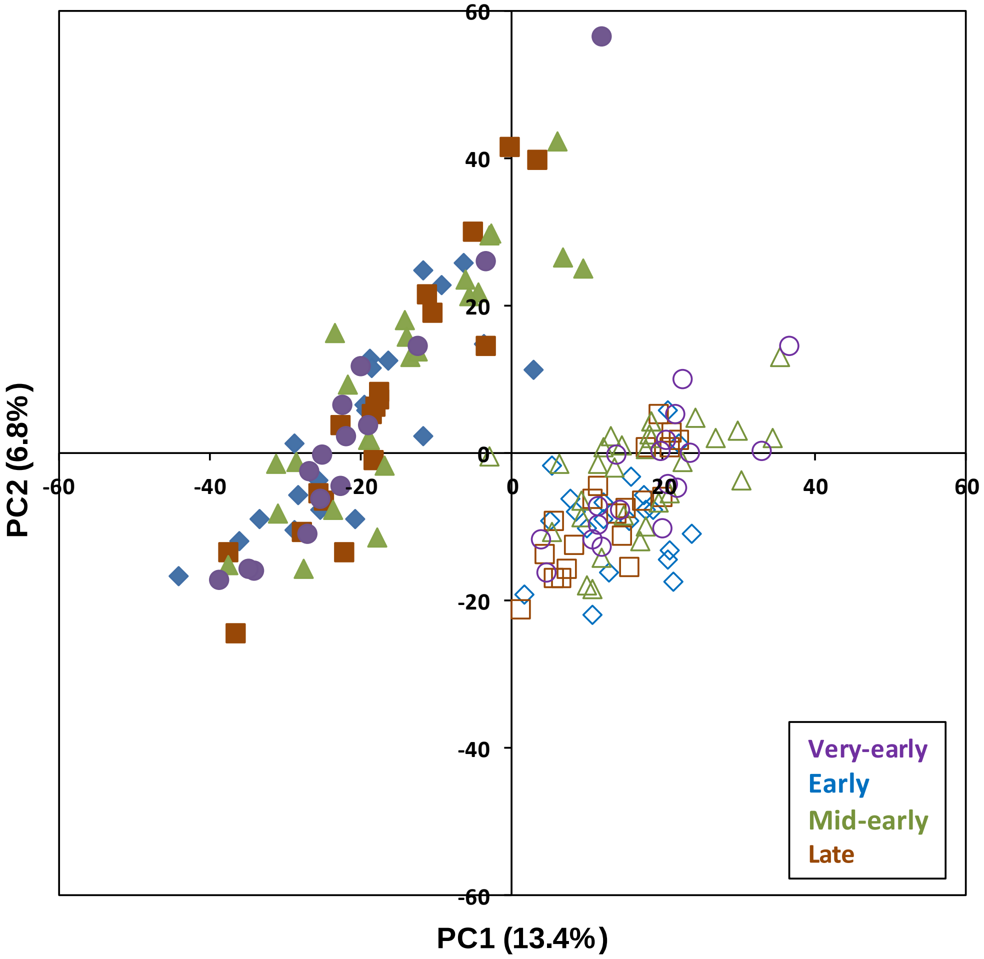

Supplementary Figure S2: Principal component analysis (PCA) of 2,868 metabolite features and starch measured in young maize leaf of 30 hybrids in the normal (full symbols) and early sowing (open symbols) conditions. PCA scores plot of the first two principal components as in Figure 1. Scores symbols correspond to the genotype silage-earliness groups defined in Table 1: Very-early, purple circle; Early, blue diamond; Mid-early, green triangle; Late, brown square.
