# Supplementary material for: Metabotyping of 30 maize hybrids under early-sowing conditions reveals potential marker-metabolites for breeding
Source: Metabolomics. 2018 Sep 26;14(10):132. doi: 10.1007/s11306-018-1427-8 (PMC6208756; doi:10.1007/s11306-018-1427-8)
Supplement: Supplementary file 8 — Changes in relative or absolute concentrations of identified or putatively-identified metabolites in maize leaf in response to ES summarized on metabolic maps. Supplementary material 8 (XLSX 166 KB) [file 11306_2018_1427_MOESM8_ESM.pdf]

Journal: Metabolomics

Title: **Metabotyping of 30 maize hybrids under early-sowing conditions reveals potential marker metabolites for breeding**

Authors: Nadia Lamari, Vanessa Zhendre, Maria Urrutia, Stéphane Bernillon, Mickaël Maucourt, Catherine Deborde, Duyen Prodhomme, Daniel Jacob, Patricia Ballias, Dominique Rolin, Hélène Sellier, Dominique Rabier, Yves Gibon, Catherine Giauffrey, Annick Moing

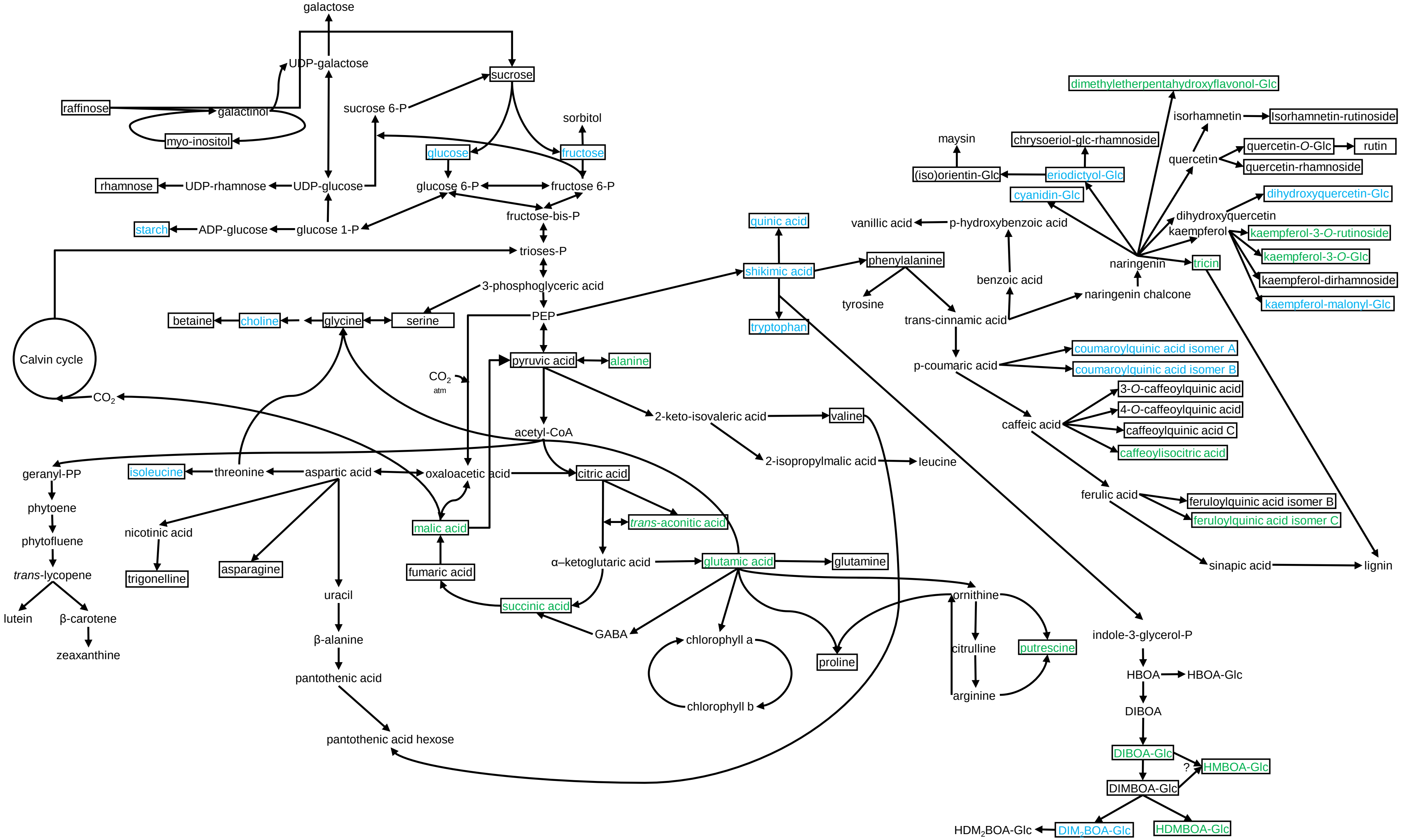

Supplementary Figure S4: Summary of the major changes in relative or absolute concentrations of identified metabolites in maize leaf in response to early sowing indicated on simplified metabolic maps. Subcellular compartmentation is not taken into account. One arrow represents one or several enzymes or enzymatic steps. Framed compounds have been quantified. They are coloured according to Student's t test results (FDR corrected *P*value<0.01) and ratio thresholds >1.2 or <0.83. Blue, significantly higher content for early sowing, Green, significantly higher content in normal sowing condition.
