# Supplementary material for: Metabotyping of 30 maize hybrids under early-sowing conditions reveals potential marker-metabolites for breeding
Source: Metabolomics. 2018 Sep 26;14(10):132. doi: 10.1007/s11306-018-1427-8 (PMC6208756; doi:10.1007/s11306-018-1427-8)
Supplement: Supplementary file 9 — Table for tentative annotation of several MS-based markers. Supplementary material 9 (PDF 134 KB) [file 11306_2018_1427_MOESM9_ESM.pdf]

Journal: Metabolomics

Title: **Metabotyping and chilling response of 30 maize hybrids** Authors: Nadia Lamari, Vanessa Zhendre, Maria Urrutia, Stéphane Bernillon, Mickaël Maucourt, Catherine Deborde, Duyen Prodhomme, Daniel Jacob, Patricia Ballias, Dominique Rolin, Hélène Sellier, Dominique Rabier, Yves Gibon, Catherine Giauffrey, Annick Moing

Supplementary Table S5: Annotation of MS markers based on LC-QTOF-MS and MS/MS data in maize leaves**.**

**Variable Name**	**RT** (min)	**Marker ion** *m/z*	**Ion type**	**Putative Molecular Formula of ion**	**Calculated m/z of ion**	**Error** (mDa)	**Error (**ppm)	**mSigma value**	**MS^2 precursor *m/z***	**LC-MS/MS Negative mode spectrum**	**Putative Name**
Silage-earliness markers											
M843T1751	29.18	843.1944	-	-	-	-	-	-	843.19 ± 1.5	Signal too low	-
M741T1917	31.95	741.2325	-	-	-	-	-	-	741.23 ± 1.5	Signal too low	-
M403T2001	33.35	403.1026	$[M-H]^-$	$C_{20}H_{19}O_9$	403.1021	0.9	2.2	57.6	403.10 ± 1.5	403.1026 $[M-H]^-$<br>328.0578 $[(M-H)-C_3H_7O_2^{\bullet}]^{\bullet-}$<br>313.0340 $[(M-H)-C_4H_{10}O_2]^-$	-
Sowing condition markers											
M713T795	13.25	713.2026	$[2M-H]^-$	$C_{30}H_{37}N_2O_{18}$	713.2047	2.1	3	76.4	713.20 ± 1.5	713.2026 $[2M-H]^-$<br>356.0958 $[M-H]^-$<br>194.0460 $[(M-H)-C_6H_{10}O_5]^-$	HMBOA-glucoside
M707T993	16.55	707.1091	$[2M-H]^-$	$C_{30}H_{27}O_{20}$	707.1101	1.0	1.4	109.6	707.10 ± 1.5	707.1091 $[2M-H]^-$<br>353.0469 $[M-H]^-$<br>351.0335$[(M-H)-2H]^-$<br>173.0100 $[(M-H)-C_9H_8O_4]^-$	Caffeoylisocitrate
M329T2126	35.43	329.0647	$[M-H]^-$	$C_{17}H_{13}O_7$	329.0667	1.9	5.9	42.1	329.07 ± 1.5	329.0653 $[M-H]^-$<br>314.0416 $[(M-H)-CH_3^{\bullet}]^{\bullet-}$<br>299.0186 $[(M-H)-2CH_3]^-$<br>271.0238 $[(M-H)-2CH_3-CO]^-$<br>227.0343 $[(M-H)-C_5H_8O_2]^-$	Tricin
M224T977	16.28	224.0554	Fragment	$C_{10}H_{10}NO_5$	224.0564	1.0	4.5	26.5	386.11 ± 1.5	386.1078 $[M-H]^-$<br>224.0560 $[(M-H)- C_6H_{10}O_5]^-$<br>196.0622 $[(M-H)- C_7H_{10}O_6]^-$	HDMBOA-glucoside
M675T639	10.65	675.1917	$[2M-H]^-$	$C_{32}H_{35}0_{16}$	675.1931	1.3	1.9	85.3	675.18 ± 1.5	675.1917 $[2M-H]^-$<br>337.0909 $[M-H]^-$<br>163.0400 $[(M-H)-C_7H_{10}O_5]^-$	Coumaroylquinate B
M447T614	10.23	447.0912	$[M-H]^-$	$C_{21}H_{19}O_{11}$	447.0933	2.1	4.7	55.3	447.09 ± 1.5	447.0912 $[M-2H]^-$<br>285.0383 $[(M-H)-C_6H_{10}O_5]^-$<br>284.0308 $[(M-H)-C_6H_{11}O_5^{\bullet}]^{\bullet-}$	Cyanidin-glucoside
M465T628	10.46	465.10	$[M-H]^-$	$C_{21}H_{21}O_{12}$	465.1038	2.1	4.4	49.0	465.10 ± 1.5	465.1021 $[M-H]^-$<br>447.0917 $[(M-H)-H_2O]^-$<br>437.1065 $[(M-H)-CO]^-$<br>303.0491 $[(M-H)-C_6H_{10}O_5]^-$<br>285.0383 $[(M-H)-C_6H_{10}O_5-H_20]^-$<br>275.0539 $(M-H)-C_6H_{10}O_5-CO]^-$	Dihydroquercetin-glucoside
