# Supplementary material for: Metabotyping of 30 maize hybrids under early-sowing conditions reveals potential marker-metabolites for breeding
Source: Metabolomics. 2018 Sep 26;14(10):132. doi: 10.1007/s11306-018-1427-8 (PMC6208756; doi:10.1007/s11306-018-1427-8)
Supplement: Supplementary file 10 — Tentative validation of silage-earliness markers in 2014. Supplementary material 10 (PDF 308 KB) [file 11306_2018_1427_MOESM10_ESM.pdf]

Journal: Metabolomics

Title: **Metabotyping of 30 maize hybrids under early-sowing conditions reveals potential marker metabolites for breeding**

Authors: Nadia Lamari, Vanessa Zhendre, Maria Urrutia, Stéphane Bernillon, Mickaël Maucourt, Catherine Deborde, Duyen Prodhomme, Daniel Jacob, Patricia Ballias, Dominique Rolin, Hélène Sellier, Dominique Rabier, Yves Gibon, Catherine Giauffrey, Annick Moing

Supplementary text 2

Tentative validation of 2013 silage-earliness markers in 2014.

Materials and Methods

The genotypes, culture conditions and leaf harvest protocol were the same in 2014 and 2013. For the second year, sowing was done on 6 May 2014 which corresponds to a normal-sowing condition. The youngest ligulated-leaf samples were harvested on 17 June 2014 for 28 genotypes common to both years.

We selected the 25 variables with VIP higher than 2.5 in the OSC-OSC-PLS of 2013 data for silage groups (Table S5) and searched for them in 2014 analytical data. For raffinose with absolute quantification data, ^{1}H-NMR quantification of raffinose was performed in 2014 as described for 2013 NMR data. For the MS-based signatures with relative quantification, the corresponding variable of each MS-based signature of 2013 analytical batch was searched in the 2014 analytical batch based on exact mass and retention time (Table A in Supplementary text 2). For raffinose, mean comparisons between the silage groups in 2014 were determined using Tukey's test ($P<0.05$). For the MS-based signatures, we calculated Pearson correlation coefficient and the corresponding *P*-value between each 2013 variable and its corresponding 2014 variable using the ls-means of 28 genotypes in the two years.

Results

Concerning raffinose, in 2014 its content followed a pattern similar to that of 2013 (Fig. I in Supplementary text 2), with a progressive increase between the very-early and late silage-earliness groups and a significant difference between the very-early and late group means (Tukey's test, $P<0.05$). Concerning the MS-based signatures, we found 17 variables out of the 25 variables in the 2014 MS-based variables (Table A in Supplementary text 2). The correlation coefficients for these 17 variables are reported in Table A with their *P*-values. For 15 out of the 17 variables, the correlation between 2013 and 2014 data was positive and significant, with 11 *P*-values lower than 0.0001. Therefore, the tendencies observed in 2013 data for silage-earliness for 25 variables seem confirmed for 16 of them including raffinose.

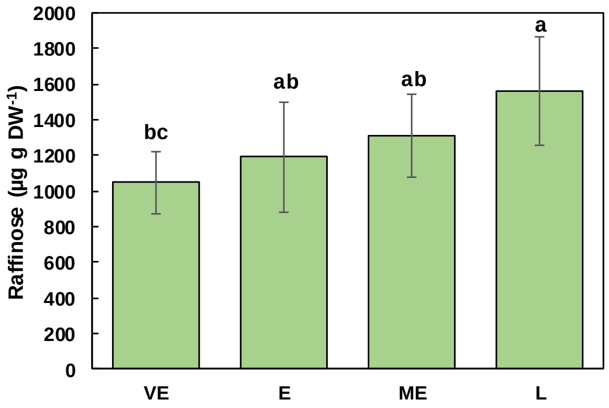

Figure I: Mean contents for each silage-earliness group in 2014 for raffinose. VE, very-early; E, early; ME, mid-early; L, late. Vertical bars represent standard deviations. Bars accompanied by the same letter are not significantly different according to Tukey's test (P<0.05).

Table A. Variable description and correlation between each of the 17 MS-based variables of 2013 (out of the 24 MS-based variables highlighted in the OSC-OSC-PLS analysis for silage-earliness in 2013) and its corresponding variable searched for in 2014 MS data. n=28.

2013			2014			2013-2014 Pearson correlation	*P*-value
Variable	Rt (s)	*m/z*	Corresponding variable	Rt (s)	*m/z*		
M247T748	748.0	247.0675	M247T741	741.3	247.0601	0.8468	<0.0001
M400T1011	1010.8	400.0874	M400T1001	1001.2	400.0879	0.6873	<0.0001
M414T806	806.0	414.0909	M414T804	803.6	414.0966	0.2654	0.17219
M478T1423	1422.9	478.1321	M478T1413	1413.1	478.1356	0.6095	0.00058
M484T765	774.3	483.9616	M484T751	751.4	484.1038	0.5000	0.00675
M486T650	650.3	486.1209	M486T651	651.0	486.1237	0.7502	<0.0001
M491T1406- dimethyletherpenta-hydroxyflavonol-glucoside	1406.1	491.1167	M491T1405	1405.1	491.1211	0.7865	<0.0001
M491T1527	1527.3	491.1180	M491T1529	1528.6	491.1207	0.8132	<0.0001
M505T1139	1139.3	505.1617	M505T1130	1130.0	505.1675	0.6175	0.00046
M513T573	572.5	513.1255	M513T608	607.9	513.1454	0.1512	0.44257
M521T1718	1717.1	521.1291	M521T1718	1717.8	521.1311	0.7013	<0.0001
M533T1587	1586.6	533.2020	M533T1585	1585.2	533.2053	0.7940	<0.0001
M535T1190	1191.2	535.1796	M535T1184	1184.2	535.1814	0.6717	<0.0001
M537T1440	1440.0	537.1562	M537T1433	1433.4	537.1609	0.5502	0.00242
M643T1261	1260.7	643.2209	M643T1261	1260.5	643.2234	0.6955	<0.0001
M669T1205	1205.1	669.1992	M669T1203	1203.4	669.2037	0.7211	<0.0001
M787T1719	1718.7	787.1705	M7871722	1722.4	787.1791	0.7523	<0.0001
