# Supplementary material for: Metabotyping of 30 maize hybrids under early-sowing conditions reveals potential marker-metabolites for breeding
Source: Metabolomics. 2018 Sep 26;14(10):132. doi: 10.1007/s11306-018-1427-8 (PMC6208756; doi:10.1007/s11306-018-1427-8)
Supplement: Supplementary file 11 — Biplots of aerial biomass with the content of each metabolite directly linked with it in Figures 4A-B. Supplementary material 11 (PDF 428 KB) [file 11306_2018_1427_MOESM11_ESM.pdf]

Journal: Metabolomics

Title: **Metabotyping of 30 maize hybrids under early-sowing conditions reveals potential marker metabolites for breeding**

Authors: Nadia Lamari, Vanessa Zhendre, Maria Urrutia, Stéphane Bernillon, Mickaël Maucourt, Catherine Deborde, Duyen Prodhomme, Daniel Jacob, Patricia Ballias, Dominique Rolin, Hélène Sellier, Dominique Rabier, Yves Gibon, Catherine Giauffrey, Annick Moing

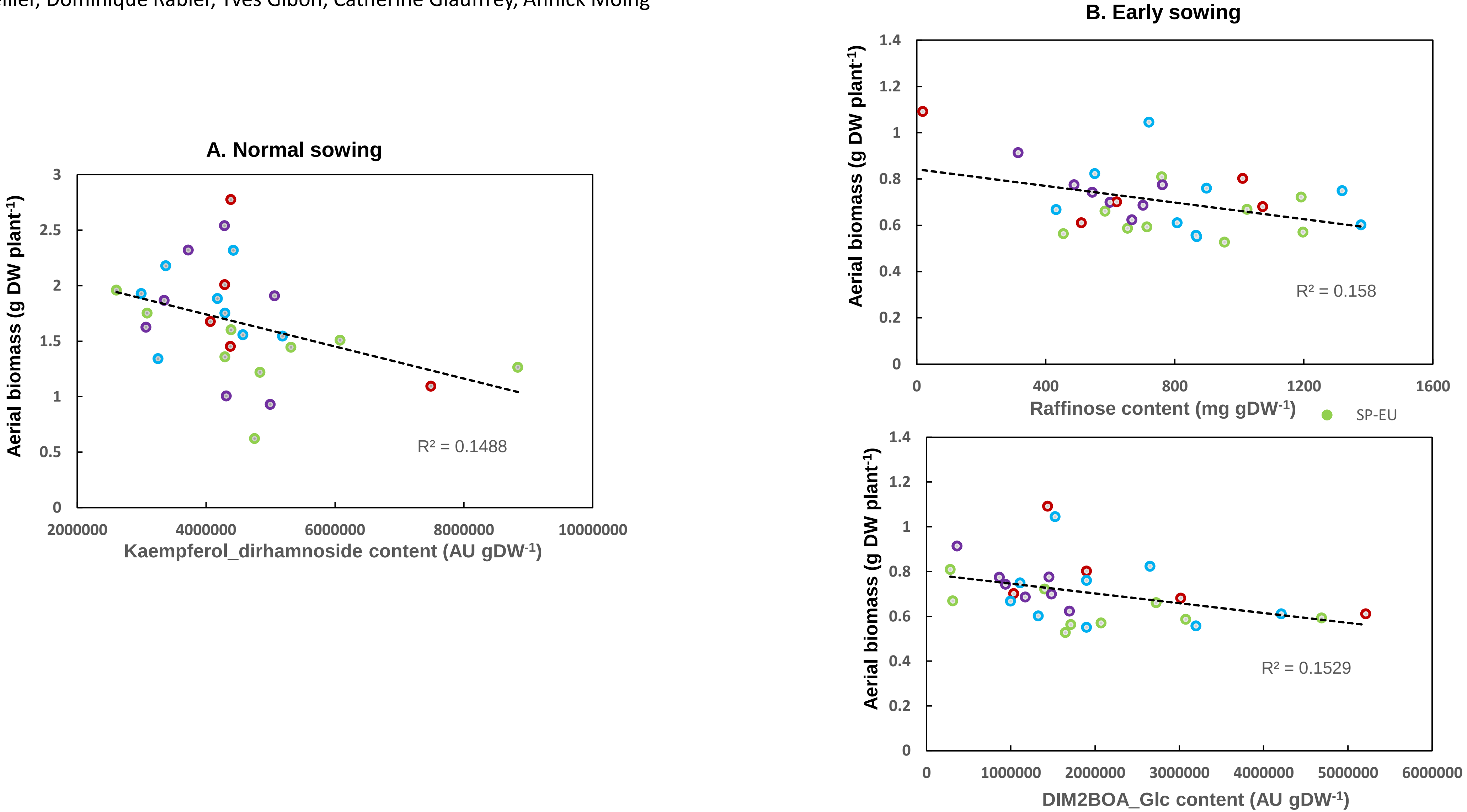

Supplementary Figure S3: Metabolites directly correlated with aerial biomass in the normal sowing or early sowing condition in Figures 4A or 4B networks. Biomass-metabolite biplots for the genotype lsmeans. Genotypes are colored according to their group (European, green; Iodent, purple; Lancaster, red; Stiff Stalk, blue). The regression line and R^2 value correspond to all genotypes.
